# Two decades of SPECT/CT – the coming of age of a technology: An updated review of literature evidence

**DOI:** 10.1007/s00259-019-04404-6

**Published:** 2019-07-04

**Authors:** Ora Israel, O. Pellet, L. Biassoni, D. De Palma, E. Estrada-Lobato, G. Gnanasegaran, T. Kuwert, C. la Fougère, G. Mariani, S. Massalha, D. Paez, F. Giammarile

**Affiliations:** 1 Rappaport School of Medicine, Israel Institute of Technology, Haifa, Israel; 2 Nuclear Medicine and Diagnostic Imaging Section International Atomic Energy Agency, Vienna, Austria; 3 Department of Radiology, Great Ormond Street Hospital for Children NHS Foundation Trust, London, UK; 4 Nuclear Medicine Unit, Circolo Hospital, ASST-Settelaghi, Varese, Italy; 5 Department of Nuclear Medicine, Royal Free NHS Foundation Trust, London, UK; 6 Clinic of Nuclear Medicine, University Hospital, Erlangen, Germany; 7 Division of Nuclear Medicine and Clinical Molecular Imaging, Department of Radiology, University Hospital, Tubingen, Germany; 8 Regional Center of Nuclear Medicine, University of Pisa, Pisa, Italy; 9 Department of Medicine, University of Ottawa Heart Institute, Ottawa, Canada; 10 Department of Nuclear Medicine, Rambam Healthcare Campus, Haifa, Israel

**Keywords:** SPECT/CT, Oncology, Endocrinology, Infection, Orthopaedics, Paediatrics, Cardiopulmonary

## Abstract

**Purpose:**

Single-photon emission computed tomography (SPECT) combined with computed tomography (CT) was introduced as a hybrid SPECT/CT imaging modality two decades ago. The main advantage of SPECT/CT is the increased specificity achieved through a more precise localization and characterization of functional findings. The improved diagnostic accuracy is also associated with greater diagnostic confidence and better inter-specialty communication.

**Methods:**

This review presents a critical assessment of the relevant literature published so far on the role of SPECT/CT in a variety of clinical conditions. It also includes an update on the established evidence demonstrating both the advantages and limitations of this modality.

**Conclusions:**

For the majority of applications, SPECT/CT should be a routine imaging technique, fully integrated into the clinical decision-making process, including oncology, endocrinology, orthopaedics, paediatrics, and cardiology. Large-scale prospective studies are lacking, however, on the use of SPECT/CT in certain clinical domains such as neurology and lung disorders. The review also presents data on the complementary role of SPECT/CT with other imaging modalities and a comparative analysis, where available.

**Electronic supplementary material:**

The online version of this article (10.1007/s00259-019-04404-6) contains supplementary material, which is available to authorized users.

## Introduction

The advent of positron emission tomography (PET) combined with computed tomography (CT), namely PET/CT, generated great excitement and anticipation in the imaging community. A publication released less than a decade after its introduction [1] envisaged a soon-to-come scenario where wide availability of positron-emitting radiopharmaceuticals would virtually replace all single-photon-emitting agents, making conventional planar and tomographic imaging obsolete. Ten years after that dismal forecast, we are witnessing an era of revived interest in single-photon imaging. The continuing evolution of technology and expertise in the field takes advantage of the full synergism between single-photon and positron emission imaging [2], while also continuing the debate regarding the optimal strategies for managing the wealth of clinically relevant information that can be obtained [3]. Hybrid imaging, including single-photon emission computed tomography (SPECT) combined with computed tomography (CT), SPECT/CT, translates molecular and metabolic information into an immediate clinical impact for a wide range of diseases. Scintigraphy is characterized by an inherent high sensitivity and negative predictive value (NPV), enhanced by SPECT. With the addition of CT, SPECT/CT further improves the diagnostic accuracy, specificity, and positive predictive value (PPV) of nuclear medicine studies. The use of SPECT/CT is rising in frequency and spreading to new clinical settings. Recent trends in sales of new nuclear medicine equipment confirm that installations of SPECT/CT devices exhibit a steep surge worldwide [4].

The value of SPECT/CT towards improved staging, prognosis and treatment planning and monitoring for a wide variety of diseases, as published up to 2008–2009, have been reviewed when SPECT/CT was just coming out of its infancy stage [5]. Over the last decade, significant advances in technology have taken place, and literature evidence has continued to accumulate. For these reasons, it was deemed appropriate to undertake an up-to-date review of the current uses of SPECT/CT, not only as a problem-solving approach, but also, and most importantly, as a diagnostic tool fully integrated into the clinical approach. More than 400 publications have been reviewed for this purpose. The complex technological issues involved in the use of SPECT/CT, such as novel radiopharmaceuticals, hardware, image acquisition protocols, quantitation, and dosimetry and radiation exposure, are beyond the scope of the current review.

## Oncology applications

SPECT/CT has found its way into most clinical scenarios in patients with malignancies who undergo tests performed with single-photon-emitting tracers. The availability of both functional and structural data has resulted in a proven synergistic effect on the diagnostic potential in the assessment of cancer. Modern SPECT/CT devices are equipped with a CT component with diagnostic capabilities. This provides the potential to enhance the value of this modality for assessment of tumours by performing a contrast-enhanced CT (ceCT) as part of the hybrid study. While currently not routinely performed or even recommended for SPECT/CT, this warrants further consideration based on the strong evidence accumulated for PET/CT. Current clinical applications of SPECT/CT in cancer need to be reviewed, keeping in mind that PET/CT alternatives are available in many centres, and choosing the most beneficial procedure for the individual patient is imperative.

### Sentinel lymph node mapping

Detection of lymphatic metastatic involvement is important in the staging, prognosis and treatment of various malignancies. Following interstitial radiocolloid injection at the site of the primary tumour, scintigraphy visualizes its lymphatic drainage. The term “sentinel lymph node” (SLN) is used to describe the first lymph node (LN) encountered by lymphatic vessels draining the primary tumour. Lymphatic drainage is complex, and SPECT/CT can achieve accurate pre-surgical SLN mapping, with further implementation into a personalized surgical approach [6].

**Fig. 1:**
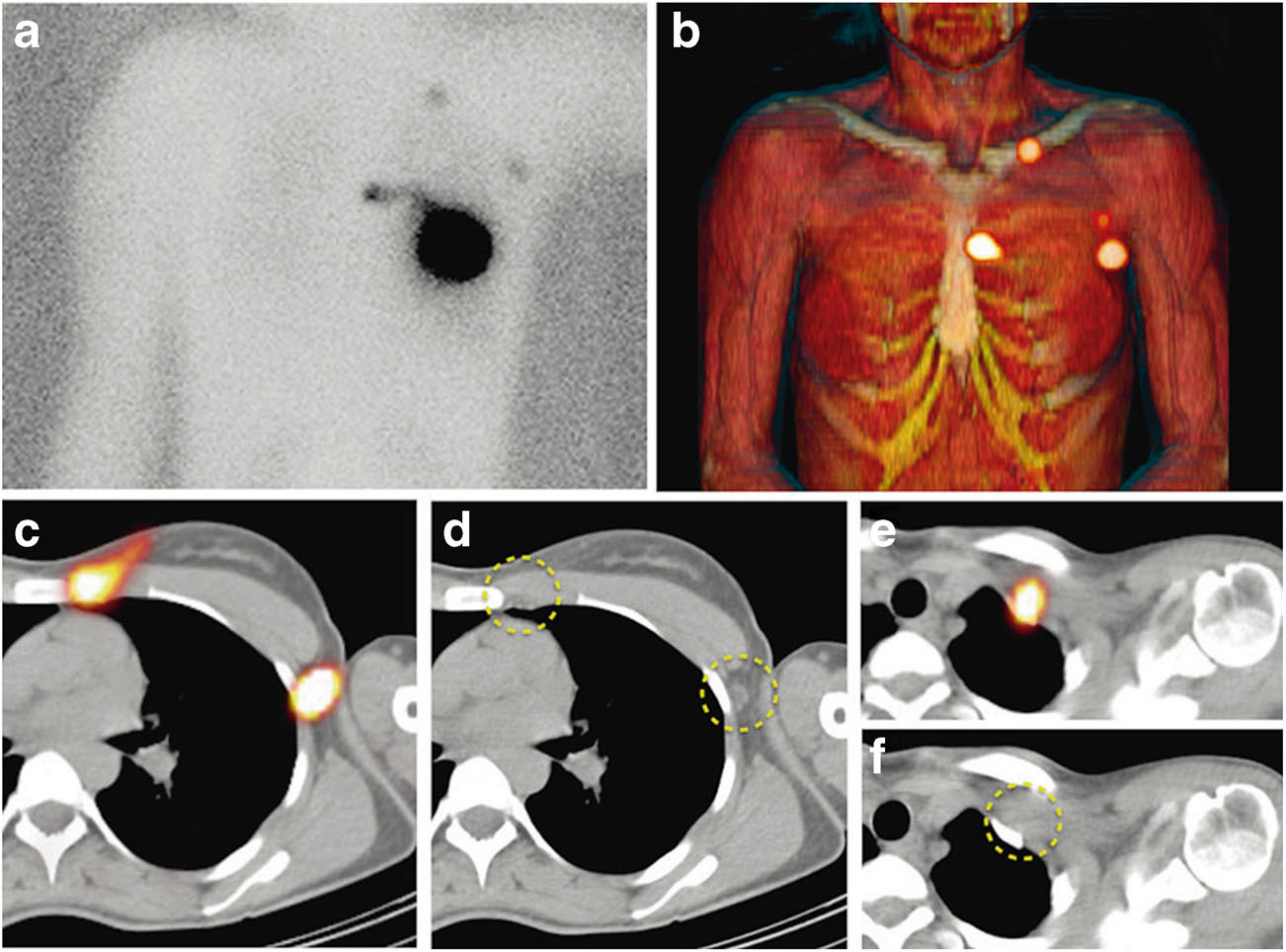
^99m^Tc-nanocolloid SPECT/CT in sentinel node lymphoscintigraphy. Preoperative sentinel lymph node (SLN) mapping in a 63-year-old woman with cancer of the left breast following intratumoral injection of ^99m^Tc-nanocolloidal albumin. Planar imaging with body contouring (**a**), with a ^57^Co flood source beneath the patient’s body, visualizes lymphatic drainage to SLNs in the left axilla, periclavicular area, and internal mammary chain. 3D surface volume rendering SPECT/CT (**b**) identifies the anatomical correlates of the SLNs localized on transaxial SPECT/CT and CT slices to the second intercostal space and level I of the left axilla, respectively (**c**, **c**), as well as behind the left clavicle (**e**, **f**), as indicated by dashed yellow circles. *[Reproduced with permission from: Giammarile F, Orsini F, Valdés Olmos RA, Vidal-Sicart S, Giuliano AE, Mariani G. Radioguided surgery for breast cancer. In: Strauss HW, Mariani G, Volterrani D, Larson SM, Eds. Nuclear Oncology – From Pathophysiology to Clinical Applications. New York: Springer; 2017:1363–1400]*

In breast cancer, where SLN mapping is well validated, a literature search yielded 41 articles on SPECT/CT, 16 of which were retained for analysis (Fig. 1, Online Table 1).

Similarly, SLN mapping has been validated in patients with intermediate-thickness melanoma. A literature search yielded 19 out of 54 articles about SPECT/CT in melanoma that were retained for analysis (Online Table 2).

In early-stage head and neck malignancies, SLN biopsy is increasingly used for treatment stratification, being associated with decreased morbidity and better outcomes as compared to elective neck dissection [7, 8]. The addition of SPECT/CT to dynamic lymphoscintigraphy in oral carcinoma revealed additional SLNs in 22% patients, providing new anatomical information in 3% of patients, for an overall detection rate for the combined approach of 98% [8, 9].

In gynaecological cancers, SLN mapping using SPECT/CT facilitates intraoperative SLN biopsy in cancer of the cervix [10, 11] and vulva [12, 13]. SPECT/CT localization of SLNs was found to be anatomically accurate in 91% of endometrial tumours, a malignancy with a low prevalence of nodal metastases [14]. One prospective study demonstrated the value of SPECT/CT in penile cancer, improving both the LN detection rate and their precise localization in drainage basins [15].

### Thyroid cancer

**Fig. 2:**
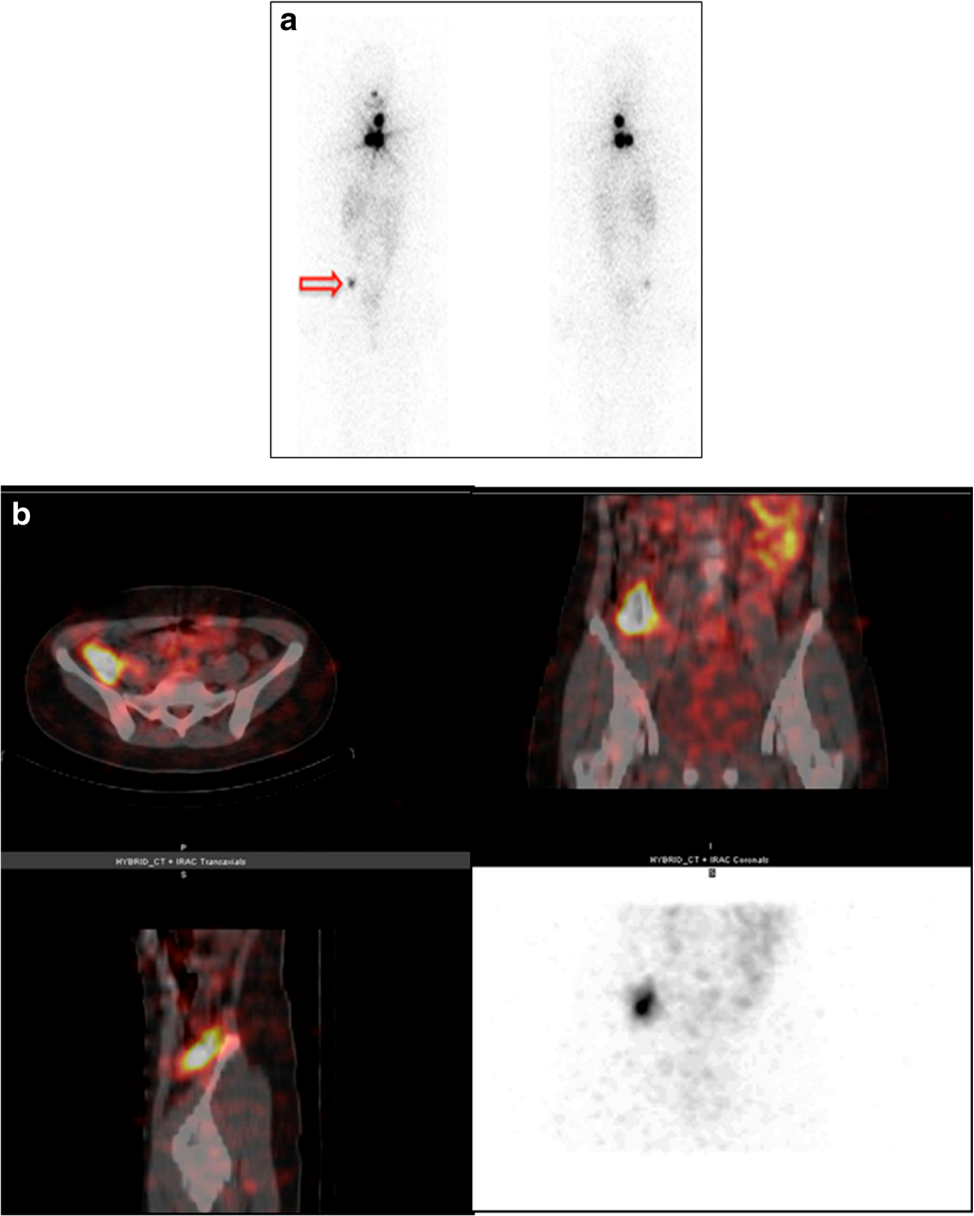
Post-ablation ^131^I-iodide SPECT/CT in differentiated thyroid cancer (DTC). A 48-year-old woman after thyroidectomy and lymphadenectomy for DTC (18 mm, infiltrating the thyroid parenchyma and capsule), with intravascular tumour emboli and nodal metastases found at surgery, stage pT1bN1bMx. The patient received 2.96 GBq ^131^I-iodide treatment. Planar post-ablation ^131^I-iodide scan (**a**) shows multiple sites of intense uptake in the neck and upper mediastinum consistent with known metastatic spread. There is an additional area of focal tracer accumulation of moderate intensity in the right pelvis (arrow). SPECT/low-dose CT slices and maximum intensity projection (MIP) image of the pelvis (**b**) locate this uptake to the lower portion of the ascending colon, consistent with physiologic tracer excretion. SPECT/CT excluded the presence of an additional malignant site in an equivocal finding detected on planar scintigraphy. *[Images provided courtesy of Drs. Paola A. Erba and Roberta Zanca, Regional Center of Nuclear Medicine, University of Pisa, Italy]*

Recent guidelines [16] present the optimal modalities for differentiated thyroid cancer (DTC) management. The selection of patients for post-surgical radioiodine (RAI) ablation is based on clinical and histopathologic risk stratification [17–20]. RAI whole-body scintigraphy (WBS) at the completion of ablation can change the risk stratification [18]. SPECT/CT provides additional information, mainly by clarifying equivocal planar findings (Fig. 2) [19]. RAI-SPECT/CT has been compared with RAI-WBS at various stages of DTC, including pre- and post-ablation, and after therapy for recurrent or metastatic disease.

Prior to ablation, RAI-SPECT/CT identified unexpected cervical nodal metastases in 30–44% and distant lesions in 4–10% of cases, leading to a change in management in 30–60% of patients, mostly receiving higher RAI treatment doses than initially planned (Online Table 3) [21–24]. After ablation, SPECT/CT was significantly more specific than WBS with or without SPECT, showing a definite incremental value in 42% of cases [25]. In multiple studies involving large numbers of patients, SPECT/CT correctly characterized over 90% of equivocal RAI foci seen on WBS, and detected additional, cervical LNs or distant metastases in 9–40% of cases. This resulted in modification of the TNM stage on average in 10%, the risk category in 35%, and planned management in up to 15% of patients [26–39]. Post-ablation RAI-SPECT/CT has also been used for radiation dosimetry estimates [40].

After RAI treatment for recurrent or metastatic DTC, SPECT/CT provided important information in 73.9% of cases and led to a change in management in 47.1% of patients [41]. SPECT/CT detected unexpected nodal neck metastases in 83.1%, lung metastases in 15.5%, and bone lesions in 2.8% of patients [42]. Pretreatment ^124^I-iodide PET/CT and post-therapy SPECT/CT were in agreement in 97% of lesions [43]. A lesion-based comparison of pretreatment [^18^F]fluorodeoxyglucose (FDG) PET/CT with pre- and post-treatment RAI-SPECT/CT and WBS showed that pretreatment SPECT/CT performed better than planar WBS or [^18^F]FDG PET/CT, while post-therapy SPECT/CT performed better than WBS but worse than [^18^F]FDG PET/CT [44]. SPECT/CT had a definite diagnostic impact in an average of 57% of DTC patients (Online Table 4). It detected unexpected sites of cervical LN or distant metastases in about 25% of patients, thus up- or down-staging over 20% and leading to change in planned management in about 25% of patients. [45–51].

### Neuroendocrine neoplasms

**Fig. 3:**
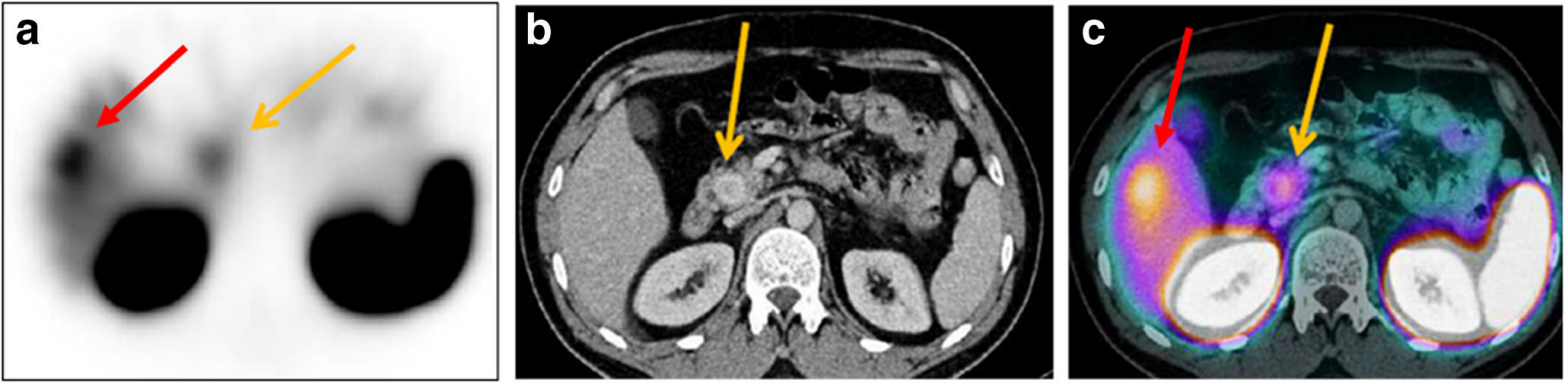
^111^In-somatostatin SPECT/CT in neuroendocrine neoplasms. A 34-year-old patient with biochemical suspicion of NEN and a lesion in the head of pancreas on CT was referred for ^111^In-somatostatin scintigraphy for staging. SPECT (**a**) shows two foci of intense tracer uptake in the upper abdomen, localized by contrast-enhanced CT (**b**) to a hypervascular primary lesion in the head of the pancreas and an isodense metastasis in the right lobe of the liver (arrows), confirmed on SPECT/CT (**c**)

Neuroendocrine neoplasms (NENs) are a heterogeneous group of tumours originating from single or clustered neuroendocrine cells, located in the gastrointestinal tract (GIT) and lungs and less commonly in the thymus, adrenal medulla, and the pituitary, parathyroid and thyroid glands. Having a nonspecific clinical presentation, NENs represent a diagnostic and therapeutic challenge. The European Neuroendocrine Tumour Society (ENETS) diagnostic and prognostic stratification criteria used in the management decision process are based on histological typing, differentiation, grading, and TNM staging. Imaging plays a fundamental role in the diagnosis, staging, treatment selection, and follow-up of NENs. Specifically, scintigraphy of tumour somatostatin receptor (SSR) expression or catecholamine uptake aims to identify functionally active lesions and has theragnostic potential. SPECT/CT has demonstrated an incremental value for assessment of NENs (Fig. 3), with a literature search yielding a total of 30 articles, 26 of which were retained for further analysis (Online Table 5). Radiolabelled octreotide scintigraphy has been used for assessment of SSR-positive NENs [52–62] with the addition, in recent years, of PET/CT with ^68^Ga-labelled somatostatin analogues. Catecholamine metabolism is assessed with ^123^I-metaiodobenzylguanidine (mIBG), ^18^F-DOPA, and potentially [^11^C]5-hydroxytryptophan (5-HTP). While the use of PET/CT is associated with higher diagnostic accuracy, better patient compliance and comfort, and lower radiation exposure, the use of SPECT/CT has a proven incremental value in the assessment of NENs (Online Table 5).

### Bone metastases

Bone metastases are associated with worse prognosis and decreased survival [63, 64]. Bone scintigraphy (BS) detects metastases in the presence of a reactive increase in bone formation. CT visualizes osseous metastases as a difference in density relative to normal tissue. Literature evidence on the diagnostic accuracy of BS in cancer is of low quality, hampered by the lack of a gold standard. Sensitivity ranges between 85% and 96% [64–66], limited by spatial resolution of planar and SPECT studies [67]. BS does not detect small osseous metastases unless they exhibit high uptake, such as in prostate cancer. Purely lytic metastases, such as in renal cancer or lymphoma, as well as predominantly lytic lesions in breast cancer, are difficult to detect by BS. These lesions can be detected on the CT component of SPECT/CT, thus enhancing the study sensitivity. Bone metastases can also be efficiently detected using FDG PET/CT, an imaging modality that plays an important role in assessing skeletal involvement in cancer, especially in the case of lesions with a predominantly osteolytic rather than osteoblastic pattern [63]. The specificity of BS for detection of bone metastases is low, since multiple benign conditions show increased radiotracer uptake, thus requiring a differential diagnosis [68]. However, since most benign conditions have a typical appearance on CT, the combined information provided by SPECT/CT adds specificity to BS in the assessment of skeletal involvement in malignant diseases.

**Fig. 4:**
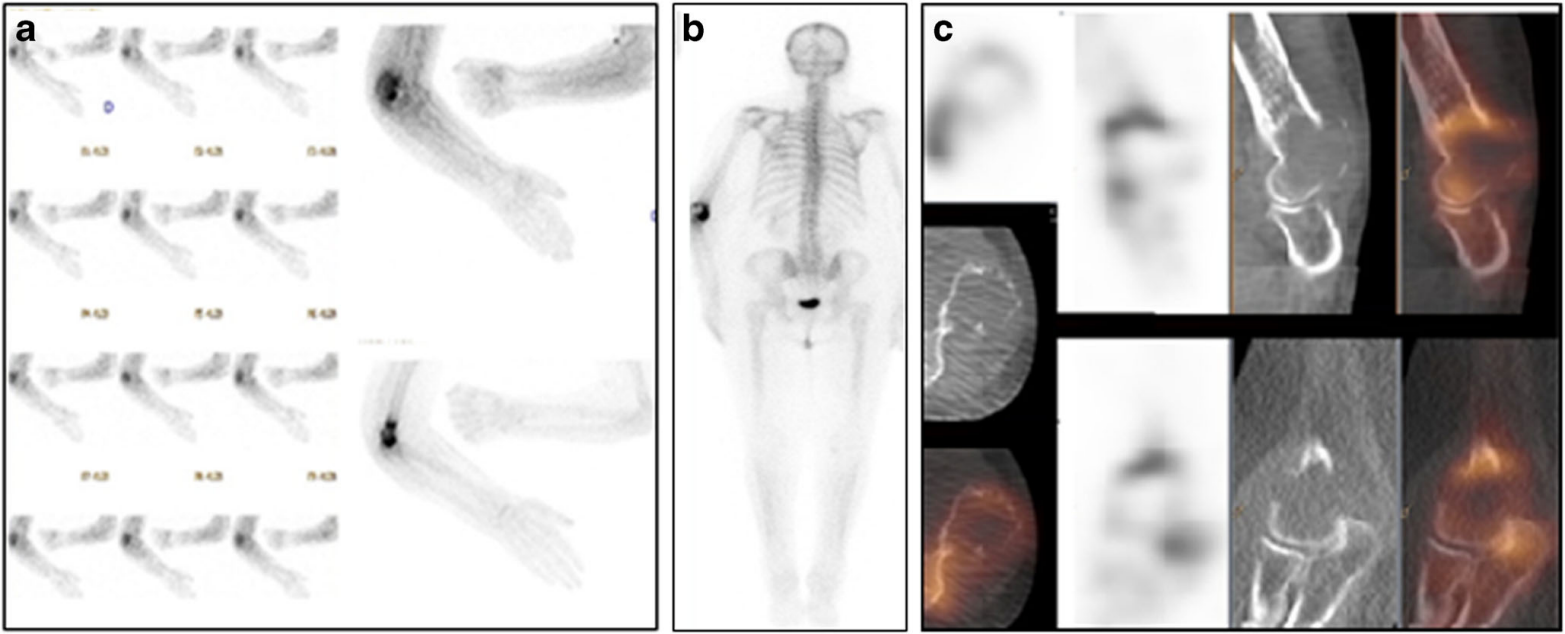
^99m^Tc-DPD bone SPECT/CT in a patient with renal cancer and bone pain. A 62-year-old patient with renal cancer was referred for evaluation of painful left elbow. Early planar scan of the elbows (**a**) shows intense hyperaemia on the left side. Delayed whole-body scintigraphy (*b*, posterior view) demonstrates intense inhomogeneous focal uptake in the distal part of the left humerus, localized by SPECT/CT (**c**) to an osteolytic metastasis seen on the CT component

A literature search yielded 104 articles about bone SPECT/CT in malignancies, of which 20 were further analysed. SPECT/CT was able to characterize most equivocal findings on BS, planar and/or SPECT, in cancer patients (Fig. 4) [69–71]. Despite the heterogeneity of the studies, the results are remarkably consistent. SPECT/CT characterized 66.7–100% of equivocal findings, for an average rate of 85.3%, in 826 lesions. A comparison of sensitivity and specificity of SPECT/CT to other modalities [69, 71–78] (Online Table 6) showed its lower performance vs. whole-body (WB)-MRI [69] and conflicting results vs. ^18^F-fluoride PET/CT [69, 77]. A new perspective for skeletal scintigraphy is WB-SPECT/CT substituting for planar BS. WB-SPECT/CT demonstrated higher sensitivity and similar specificity to WB-planar and one-field-of-view (FOV)-position-targeted SPECT/CT [79], but with only limited incremental diagnostic value for 2-FOV vs. 1-FOV bone SPECT/CT [80]. It seems reasonable to predict that WB-SPECT/CT will be the future of BS in cancer patients, in particular when fast acquisition protocols become widely available [81].

### Prostate cancer

**Fig. 5:**
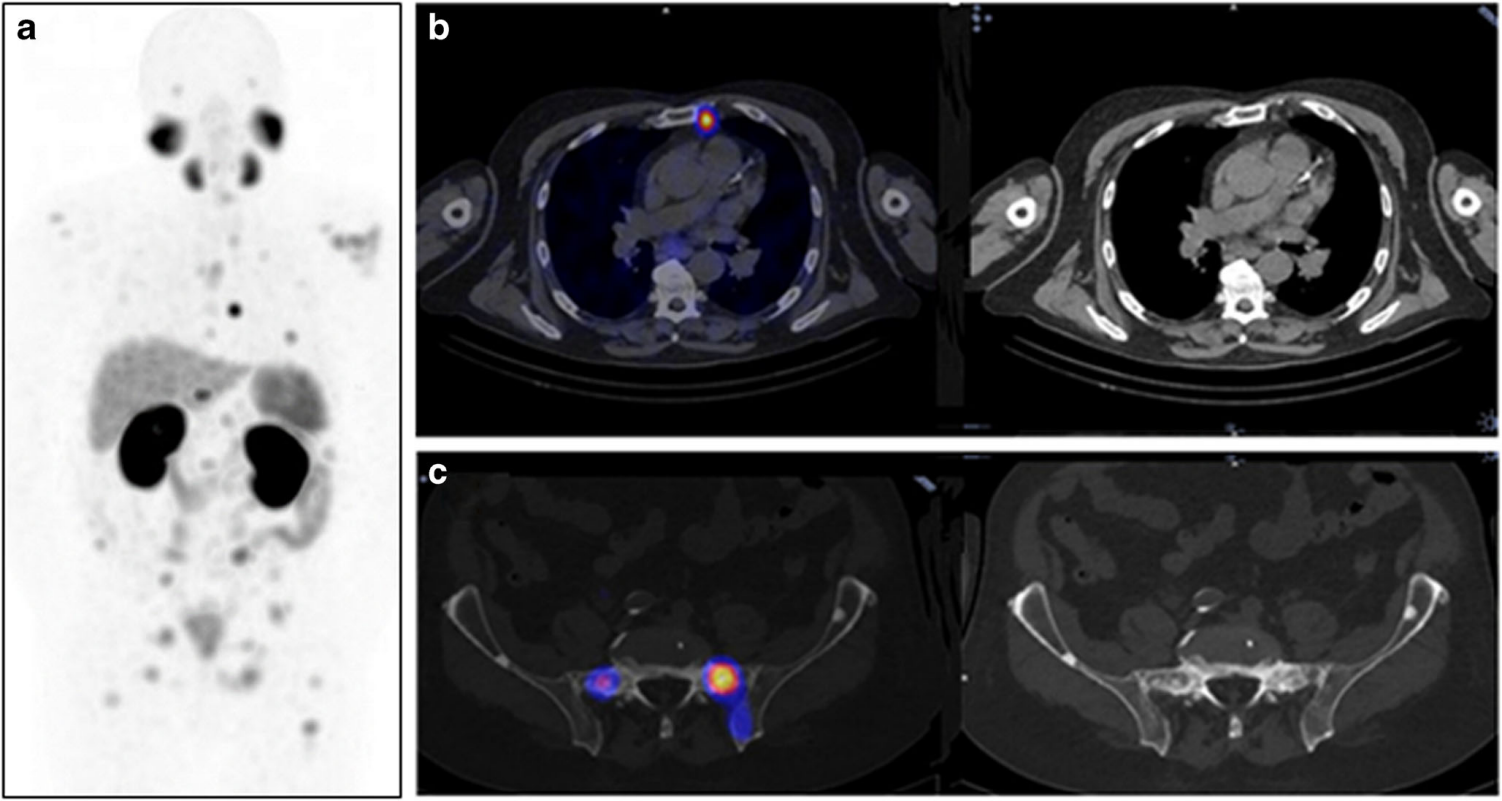
^99m^Tc-MIP-1404 PSMA-SPECT/CT in a patient with prostate cancer. An 81-year-old man with prostate cancer (pT4, N2, M0, G3) with biochemical recurrence diagnosed by increased serum PSA levels from 6.59 ng/mL to 17.34 ng/mL over a 3-month interval, was referred for restaging. Whole-body SPECT-MIP (**a**) shows multiple sites of focal abnormal tracer uptake above and below the diaphragm. Transaxial SPECT/CT and CT slices at the level of the upper thorax (**b**) demonstrate the presence of a left internal mammary chain lymph node metastasis. Transaxial SPECT/CT and CT slices at the level of the upper pelvis (**c**) show multiple, partially MIP-1404-avid osteoblastic lesions in the bone, consistent with functionally active and inactive skeletal metastases

The development of PET radioligands directed against the prostate-specific membrane antigen (PSMA) has revolutionized the diagnostic workup of prostate cancer [82, 83]. ^99m^Tc-labelled PSMA-ligand agents have also recently been developed [84–88]. SPECT/CT with ^99m^Tc-MIP 1404 (PSMA-ligand subtype) enabled the detection of small LNs or additional metastases (Fig. 5). SPECT/CT achieved sensitivity of up to 97% for diagnosis of primary prostate cancer [89, 90]. In a group of 225 patients with biochemical relapse, the detection rate correlated with prostate-specific antigen (PSA) levels [91]. PSMA-SPECT/CT also demonstrated higher sensitivity than BS with SPECT/CT and MRI for the detection of skeletal metastases in patients with biochemical recurrence of prostate cancer [92]. However, a small comparative study in 14 patients showed the superiority of ^68^Ga-PSMA-PET/CT over ^99m^Tc-HYNIC PSMA-SPECT/CT for detection of malignant sites [93].

### Transarterial radioembolization

The liver represents a frequent site of primary cancer and metastatic disease. Transarterial radioembolization (TARE) uses percutaneous intra-arterial techniques to inject micron-sized embolic particles, ^90^Y- or more recently ^166^Ho-microspheres, for treatment of malignant liver lesions. Beta irradiation favours destruction of the tumour cells surrounding microvessels with high radioactive particle concentration. The supply of blood to hepatic tumours derives mainly from the arterial circulation, and therefore, delivery of radioactive compounds into the hepatic artery can achieve highly selective tumour uptake. The disadvantages of TARE are related to potential inadvertent delivery or shunting.

A pre-therapy angiographic evaluation combined with scintigraphy following intra-arterial ^99m^Tc-labelled albumin macroaggregate injection maps the tumour-feeding vessels, quantifies potential liver-to-lung shunting, and can detect the presence of blood reflux to the bowel, stomach, or pancreas. A pre-therapeutic SPECT/CT can better assess intra- and extrahepatic distribution of the radiotracer and can be used as an adjunct to calculate the therapeutic dose. After ^90^Y-microsphere administration, post-therapeutic bremsstrahlung SPECT/CT can verify the sphere distribution and enable post-treatment dosimetry. A literature search yielded 15 of 74 articles, focused mainly on dosimetry, that were retained for analysis (Online Table 7).

## Non-oncologic applications

### Benign skeletal conditions

**Fig. 6:**
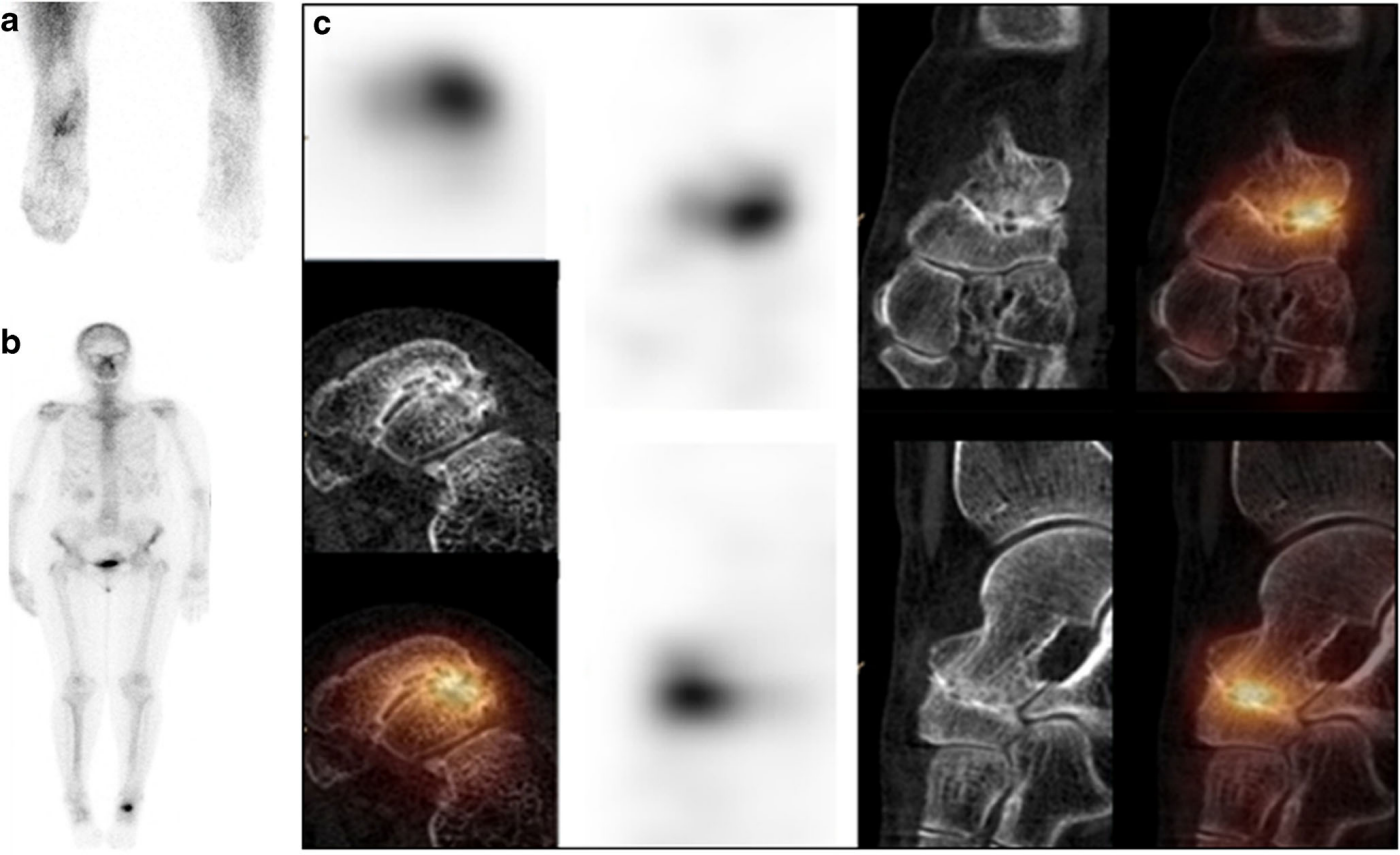
^99m^Tc-MDP bone SPECT/CT in a patient with a painful left foot. A 70-year-old patient with persistent pain in the left foot was referred for bone scintigraphy (BS) for detection of the pain generator. Early planar scan of the feet (**a**) and delayed whole-body scan (**b**) show a focus of hyperaemia and intense tracer uptake in the posterior aspect of the left tarsal region, localized by SPECT/CT (**c**) to severe degenerative changes in the left talonavicular joint seen on the CT component

^99m^Tc-methylene diphosphonate (MDP) bone SPECT/CT plays a pivotal role in the assessment of musculoskeletal (MSK) diseases, including in patients with chronic pain or with inconclusive cross-sectional imaging results (Online Table 8), providing functional and localization information and identifying specific structural patterns. SPECT/CT has improved the diagnostic accuracy of BS in trauma and rheumatic diseases such as occult fractures, inflammatory arthritis, and spondyloarthropathies (Fig. 6) [94–96].

In patients with chronic low back pain (LBP), SPECT/CT has been used to guide therapy [97–99] and to assess complications following spine surgery [100–103]. In patients with recurrent pain following lumbar arthrodesis, SPECT/CT was highly sensitive and specific for exclusion of screw loosening [102]. In pelvic girdle pain and sacroiliac joint (SIJ) dysfunction, SPECT/CT diagnosed SIJ incompetence with sensitivity of 95%, specificity of 99%, PPV of 99%, and NPV of 94% [104].

Diagnosis of hand and wrist pain by BS is challenging because of the complex regional anatomy. SPECT/CT can detect post-traumatic bone remodelling in occult fractures, often missed by other imaging tests [105–107]. SPECT/CT arthrography has been used for assessment of the scapholunate and lunotriquetral ligament or the triangular fibrocartilage complex [108]. In patients with nonspecific regional pain, SPECT/CT has shown a higher lesion detection rate than X-ray and planar BS [109] and higher specificity than MRI [110].

Detecting the source of pain following hip or knee replacement is not straightforward. X-ray is the initial test, often followed by BS to confirm or exclude septic or aseptic loosening. Increased tracer uptake identified on bone SPECT/CT in patients with total hip arthroplasty was shown to correlate significantly with symptoms [111, 112]. While BS is hampered by nonspecific tracer uptake, the CT component of SPECT/CT can identify pain generators such as osteolysis, fracture, calcifications, and joint effusion. SPECT/CT demonstrated higher diagnostic accuracy in evaluating aseptic and septic loosening of hip and knee prostheses as compared with three-phase BS and SPECT [113–115].

SPECT/CT was used to evaluate bone viability after arthroplasty and was then compared with MRI. The two techniques were complementary in the differentiation between viable and nonviable tissue [116]. In patients following knee replacement, SPECT/CT identified typical patterns in patella-femoral disorders, further improving the management of symptoms [117, 118]. After reconstruction of the anterior cruciate ligament, SPECT/CT identified bone remodelling, graft incorporation, or insufficiency [119, 120]. SPECT/CT has also been useful in the follow-up after realignment treatment, osteotomies, and unloader devices or insoles.

Assessment of foot and ankle pain is challenged by regional anatomical complexity. SPECT/CT has been used in the diagnosis of fractures, infection, pseudoarthrosis, accessory sesamoid bones, tarsal coalition, and osteochondrosis dissecans [121]. SPECT/CT and MRI provided comparable diagnostic yield in painful lesions in the ankle and foot [122]. SPECT/CT of the foot was useful in the assessment of misaligned hindfoot [123] and in characterizing impingement syndromes and soft tissue (ST) pathology in this region [124, 125]. SPECT/CT of the skull was superior to BS for the diagnosis of active condylar hyperplasia [126].

### Infection

While diagnosis of an infectious process is based on clinical and laboratory data, localization can be difficult. Infection-seeking tracers labelled with single-photon-emitting radionuclides include autologous leukocytes [white blood cells (WBC)] labelled with ^99m^Tc-hexamethylpropyleneamine oxime (HMPAO) or ^111^In-oxine [127] and, to a lesser extent, radiolabelled antibiotics, antibodies [128, 129], and ^99m^Tc-ubiquicidin 29-41 [130]. ^67^Ga-citrate is still used in a few scenarios such as osteomyelitis (OM) of the spine or sternum [131–134]. SPECT/CT enables both early diagnosis of infection and precise localization. Although there has been a recent shift in infection imaging towards [^18^F]FDG-PET/CT [135], SPECT/CT is a valid alternative.

SPECT/CT optimizes the diagnosis of clinically suspected MSK infections and localization of known processes. This is useful in cases when bone involvement has to be proven or excluded in the presence of soft tissue infection (STI) or for assessing the extent of OM in a complicated anatomical region such as in post-surgical alterations or close to implanted medical devices. Initial studies including mixed patient populations have reported that SPECT/CT with ^111^In- or ^99m^Tc-labelled WBCs or ^67^Ga-citrate had high performance indices in one third of cases [128, 131, 136–138]. A total of 24 papers were retrieved from a literature search and retained for further analysis (Online Table 9).

**Fig. 7:**
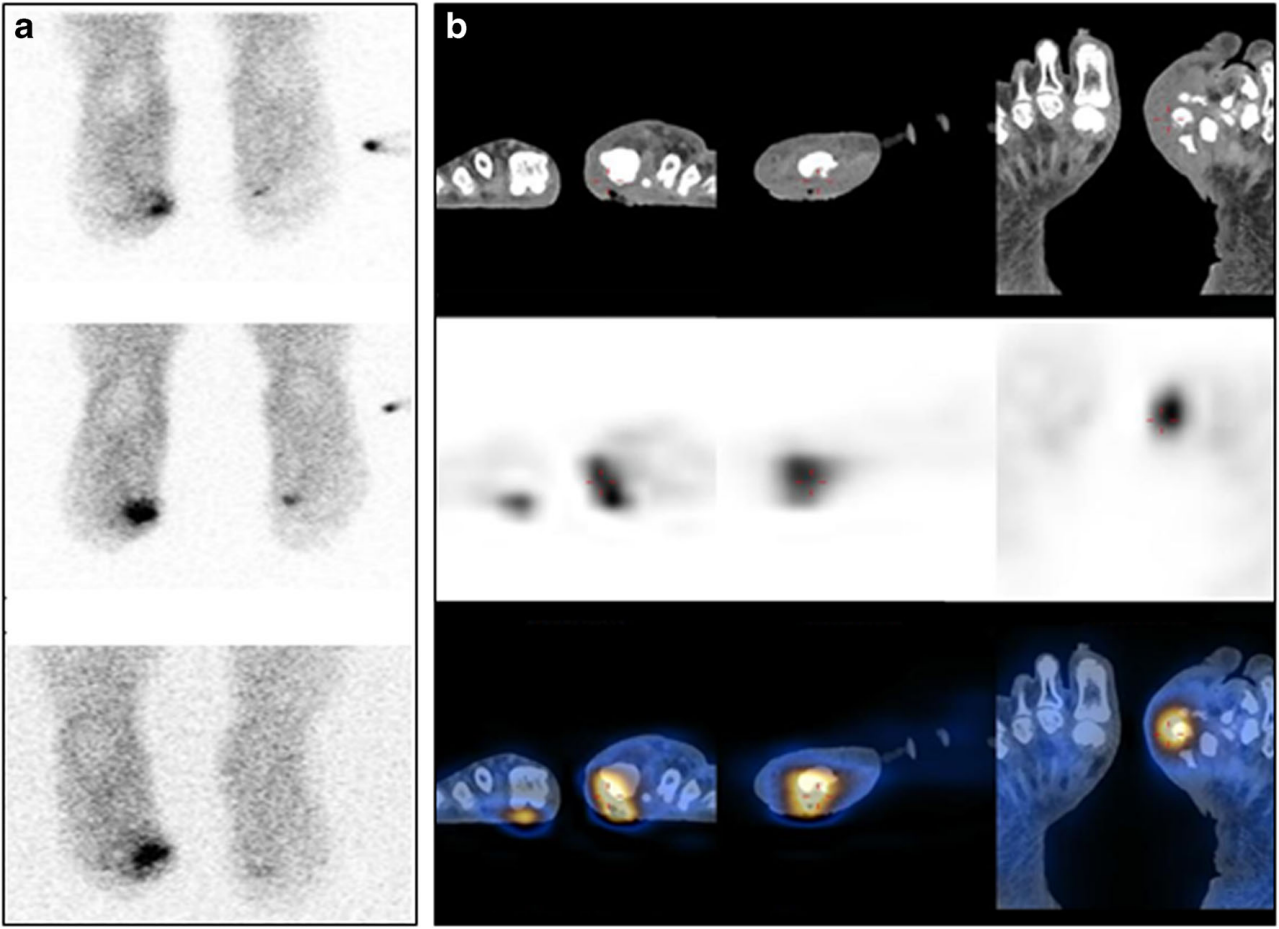
^99m^Tc-HMPAO-labelled leucocyte SPECT/CT in a patient with diabetic foot. A 43-year-old man with diabetes mellitus and an infected wound in the medial aspect of the left forefoot was referred for ^99m^Tc-HMPAO-leucocyte scintigraphy for suspected osteomyelitis. Planar scans (**a**) performed at 1 h (top), 4 h (center), and 24 h (bottom) after tracer injection show a focal area of uptake at the base of the first left digit, increasing in intensity in its superior aspect. SPECT/CT (**b**) locates this uptake to fragments of the first left metatarsal bone showing areas of cortical erosion and sclerosis, as well as to the adjacent deep wound and surrounding edematous soft tissue, consistent with osteomyelitis in addition to the soft tissue infection

OM has to be considered in any diabetic patient with chronic non-healing wounds, mainly in the feet. Studies reported that WBC scans confirmed the infection but SPECT/CT detected or excluded OM adjacent to STI in more than 50% of patients with diabetic foot [139–141], increasing specificity and PPV (Fig. 7) [142, 143]. WBC-SPECT/CT was superior to [^18^F]FDG-PET/CT [139] and similar to MRI [143] in the assessment of diabetic foot, with high sensitivity and NPV, but lower specificity and PPV at the end of antibiotic therapy [144, 145].

^67^Ga-citrate SPECT/CT demonstrated high diagnostic accuracy for spondylodiscitis, similar to MRI [132] but inferior to [^18^F]FDG-PET/CT [134]. ^111^In-diethylenetriaminepentaaceticacid (DTPA)-biotin SPECT/CT was also used for localization of spinal infection and for tailoring of therapy [146].

Differentiating aseptic loosening of a prosthetic joint from infection defines the treatment strategy. The performance indices of ^99m^Tc-WBC SPECT/CT reached 93% but were somewhat lower than for ^99m^Tc-labelled antigranulocyte antibodies [129, 147].

SPECT/CT localized foci of infection to the jaw or other bones in the base of skull [148, 149] and diagnosed OM in cases with malignant otitis externa [150]. Dual-isotope ^99m^Tc-MDP bone and ^111^In-oxine-WBC SPECT/CT provided high diagnostic confidence for evaluation of infected pelvic pressure sores [151].

STI has nonspecific clinical presentations and requires extensive diagnostic workup [131]. In this setting, SPECT/CT could be useful in vascular graft infection [131, 152–154], infectious endocarditis [155, 156], infection of cardiac implantable electronic devices [157, 158], and fever of unknown origin [131, 159, 160]. Eleven papers were retrieved from a literature search and retained for further analysis (Online Table 10).

### Parathyroid diseases

^99m^Tc-sestamibi (MIBI) SPECT/CT is used in the workup of patients with hyperparathyroidism (HPT). Variable acquisition protocols are used, including single-tracer dual-phase studies and subtraction imaging following ^99m^Tc-pertechnetate or ^123^I-iodide administration. Timing of SPECT/CT early, late, or twice during imaging has been described [161–164].

**Fig. 8:**
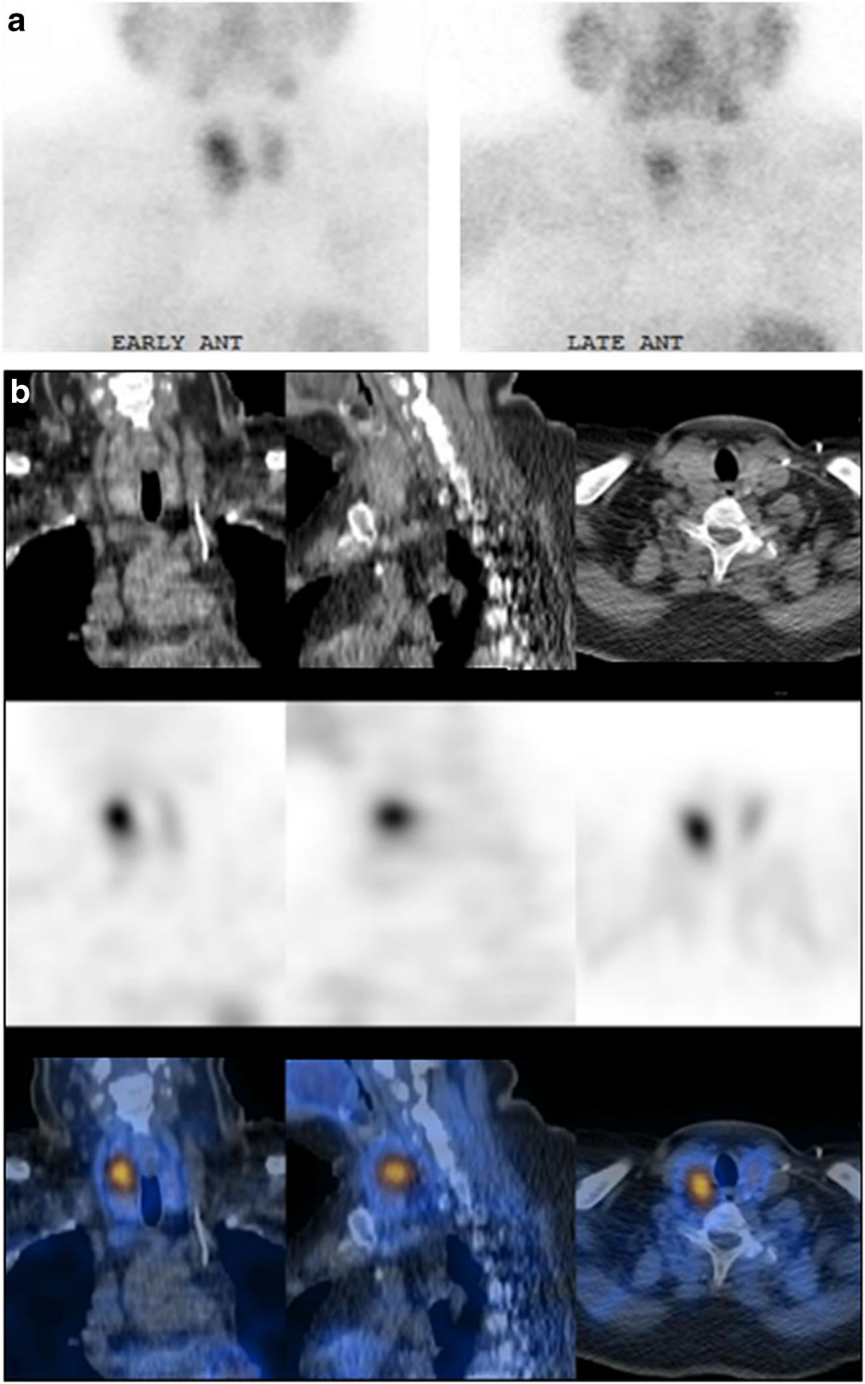
^99m^Tc-Sestamibi SPECT/CT for localization of parathyroid adenoma (PTA). A 57-year-old woman with laboratory evidence of primary hyperparathyroidism was referred for localization of PTA. Planar scintigraphy (**a**) shows an early area of increased focal uptake at the upper pole of the right thyroid lobe (left), with washout of the tracer from adjacent thyroid tissue on late images (right). SPECT/CT (**b**) performed 1 h after tracer injection localizes this focal uptake to a 16-mm nodule behind the thyroid gland, consistent with a PTA

The recent introduction of minimally invasive surgery for parathyroid adenoma (PTA) underscores the need for precise functional and topographic information provided by SPECT/CT. The main current indication for parathyroid scintigraphy is preoperative localization of PTAs (Online Table 11, Fig. 8). The detectability of PTA by SPECT/CT ranged from 90 to 96% [165, 166], particularly helpful for small lesions less than 10 mm in diameter [54, 166] or weighing less than 210 mg [167]. ^99m^Tc-MIBI SPECT/CT results were found to correlate with serum parathyroid hormone (PTH) and calcium levels [162, 168]. SPECT/CT improved localization of PTAs in 8–39% patients [169, 170], with a sensitivity range of 83–97%, specificity of 89–96%, PPV of 94–97%, and NPV of 85% [161, 163, 167, 171–173]. SPECT/CT correctly localized both ectopic PTAs and residual lesions in patients with prior neck surgery [170]. ^99m^Tc-MIBI SPECT/CT has led to a reduction of up to 50% in the duration of surgery [166, 172, 173]. Positive SPECT/CT is a good criterion for defining patient eligibility for surgery [174] and for surgical procedure planning, particularly in the presence of thyroid disease such as multinodular goitre [172]. ^99m^Tc-MIBI SPECT/CT localization of a PTA was shown to be superior to both SPECT and ultrasound (US) [54, 165, 167, 171, 173] but not to multiphase 4D CT, which added information, mainly in non-^99m^Tc-MIBI-avid lesions [175, 176]. In cases resistant to medical treatment, patients with secondary HPT are referred for parathyroidectomy, and preoperative ^99m^Tc-MIBI SPECT/CT improved surgical outcome [177–179]. A total of 23 papers retrieved through a literature search were retained for further analysis.

### Lung disorders

Ventilation and perfusion (V/Q) imaging is routinely used in the workup of patients with suspected pulmonary emboli (PE) [180]. In planar V/Q scans, distinction of anatomical segments is challenging, and it is difficult to determine the extent of embolic involvement [181–183].

**Fig. 9:**
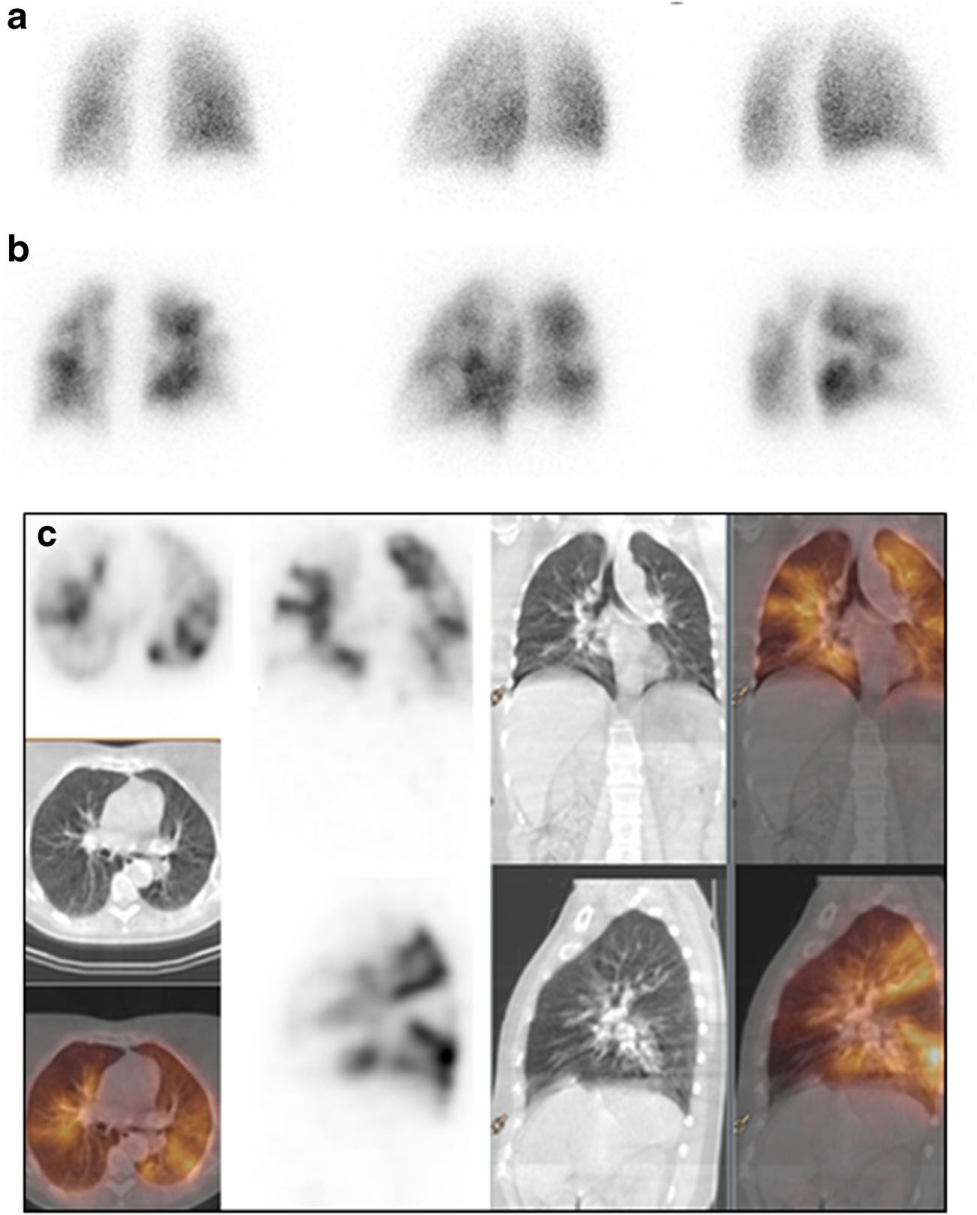
Lung SPECT/CT, perfusion study in suspected pulmonary embolism (PE). A 60-year-old patient with clinical suspicion of pulmonary embolism was referred for pulmonary ventilation/perfusion scintigraphy. Planar scintigraphy selected posterior and posterior oblique views) shows normal ventilation (**a**), in the presence of multiple segmental and subsegmental perfusion defects in both lungs (**b**). SPECT/CT shows no corresponding abnormal parenchymal changes on the CT component of the study (**c**). The patient was diagnosed with a bilateral pulmonary embolism

CT pulmonary angiography (CTPA) has gradually replaced V/Q scans for PE, being widely available and having high sensitivity and specificity. Nevertheless, CTPA is limited by technical artefacts, contrast allergy, or poor renal function [184, 185]. V/Q SPECT has improved sensitivity over planar scintigraphy, but has lower specificity than CTPA [185–191]. V/Q SPECT/CT is typically performed with low-dose CT for purposes of anatomical localization and attenuation correction (AC) [192]. The addition of CT increased specificity (Fig. 9), comparable to CTPA, and characterized abnormalities seen on SPECT in the context of lung comorbidities [189, 193]. In a comparison of V/Q SPECT, SPECT/CT, perfusion-only SPECT/CT, and CTPA for detection of PE, V/Q SPECT/CT achieved sensitivity and specificity of 100% [189, 192].

V/Q SPECT/CT has also been preliminarily described in non-PE applications including preoperative quantification of lung function, defining radiotherapy fields, and assessing regional changes in asthma, emphysema, or interstitial lung disease [192, 194–207].

### Cardiology

Coronary artery disease (CAD) is the number one cause of cardiovascular morbidity and mortality. Current guidelines recommend that patients with low pretest probability for stable CAD undergo cardiac computed tomography angiography (CCTA), which has a high NPV, while patients with high pretest probability should be referred for invasive coronary angiography (ICA). The intermediate-risk group, comprising the majority patients, needs further assessment to define the haemodynamic significance and quantify ischaemia in addition to assessing the degree of stenosis.

**Fig. 10:**
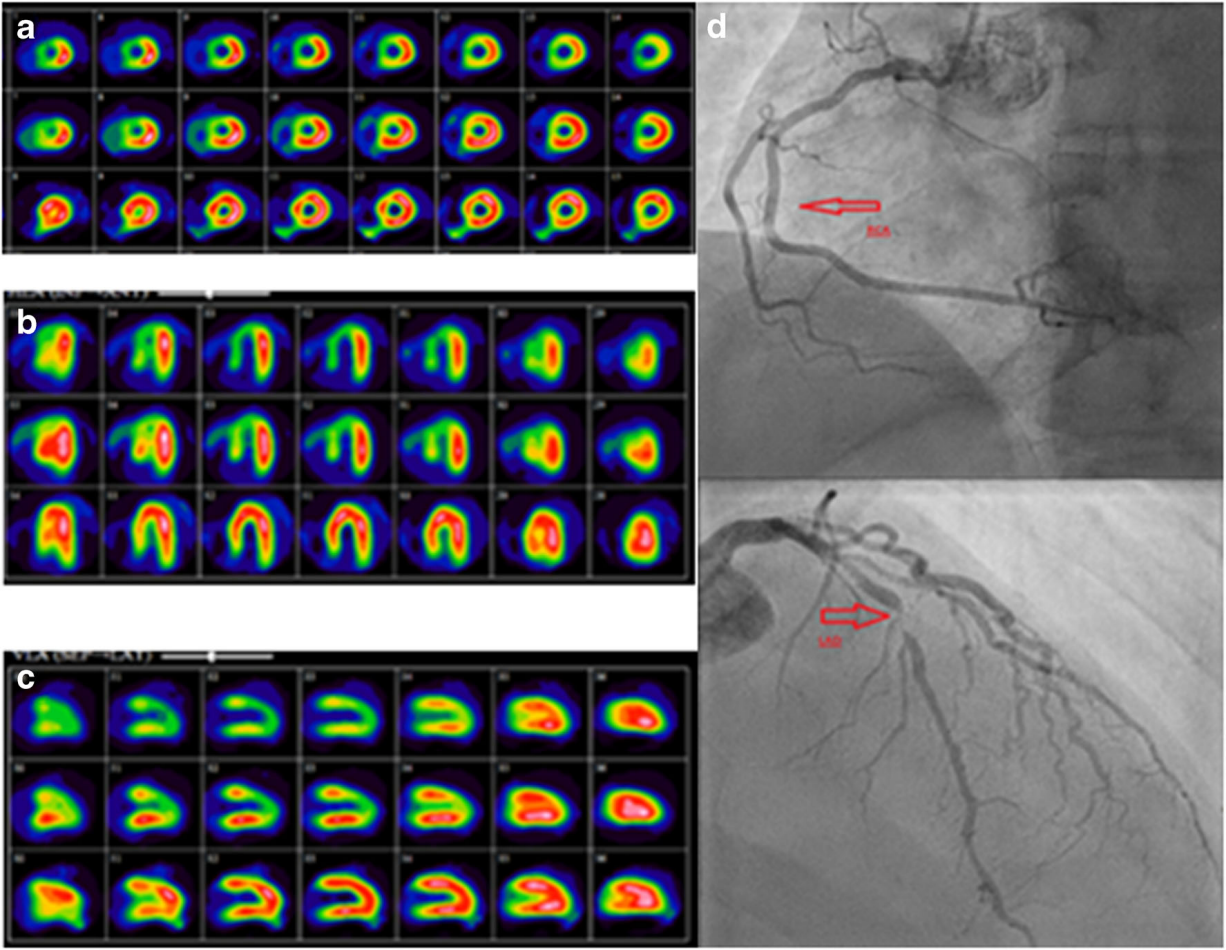
^99m^Tc-tetrofosmin cardiac SPECT/CT for attenuation correction. A patient presenting with chest pain and with family history of CAD was referred for a myocardial perfusion SPECT/CT using a single-tracer one-day stress–rest protocol with ^99m^Tc-tetrofosmin. CT AC was performed for the stress study. MPI-SPECT short (**a**), horizontal (**b**), and vertical (**c**) long axis show moderate reduction in tracer uptake in the anteroseptal wall sparing the anterobasal segment, and in the inferior wall and the apex on the non-corrected images at stress (first row), normalizing at rest (third row), suggests the presence of moderate ischemia in the territories of the left anterior descending (LAD) and right coronary (RCA) arteries. AC stress images (second row) confirm the presence of decreased uptake in the territory of the LAD only (the anteroseptal wall and apex), while correcting the attenuation artefact caused by the diaphragm in the inferior wall. The findings are consistent with a large area of moderate ischemia in the territory supplied by the LAD. Coronary angiography (**c**) demonstrates normal RCA (top, arrow) and a critical lesion in the proximal LAD (bottom, arrow)

Myocardial perfusion imaging (MPI) SPECT using ^99m^Tc-labelled tracers or ^201^Tl-chloride has been validated for diagnosis, risk stratification, and prognosis of CAD [208]. AC algorithms utilizing CT and iterative image reconstruction techniques have improved the image quality and diagnostic accuracy of SPECT (Fig. 10) [209]. MPI-SPECT with vs. without AC show sensitivity of 89% vs. 87% and specificity of 81% vs. 73%, respectively [210]. SPECT/CT used for AC improved diagnostic confidence in the interpretation of stress-only MPI studies, thus reducing patient radiation exposure following the implementation of this protocol [211].

The CT component of SPECT/CT can be also used for coronary artery calcium score (CAC) measurements without significantly increasing the radiation exposure to the patient. SPECT/CT calcium scanning is becoming a routine part of MPI, with superior diagnostic and prognostic value [212, 213].

CCTA has high diagnostic accuracy for stenosis in native coronary arteries [214–219]. SPECT/CT systems equipped with components that enable sufficient resolution to perform CCTA are now available. SPECT/CCTA led to similar patient management when compared to ICA [220].

When combining MPI with CT, the patient is exposed to additional radiation varying from 0.5 to 1.0 mSv for CT-AC. Absorbed doses for CAC and CCTA depend on the device and protocol used, estimated to be below 1 mSv for CAC measurements and between 2 and 5 mSv for CCTA, with latest-generation CT scanners even below 1 mSv [221, 222]. A total of 18 papers on cardiac SPECT/CT for AC, CAC measurements, or in combination with CCTA were retrieved by a literature search and retained for further analysis (Online Table 12).

### Neurology

**Fig. 11:**
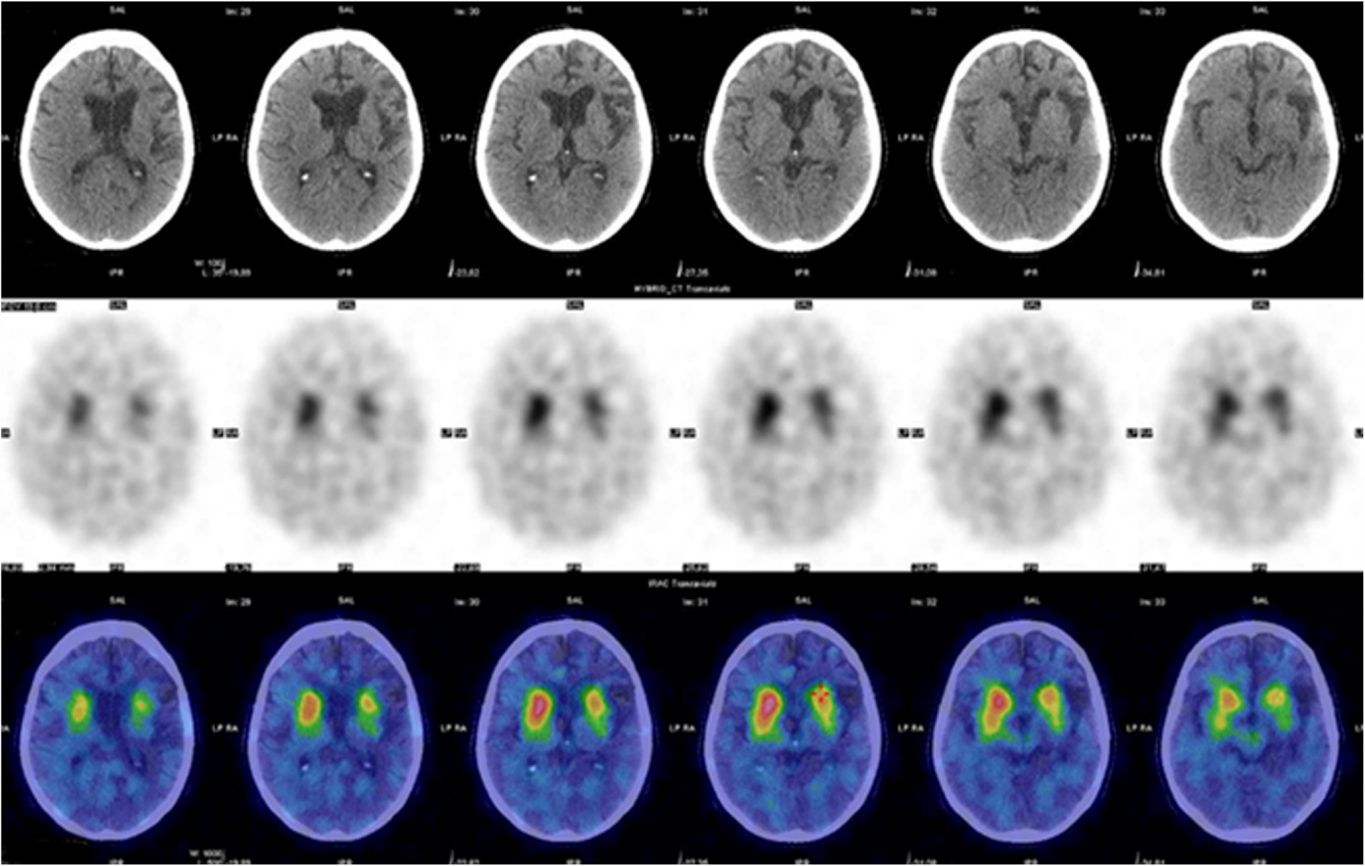
^123^I-FP-CIT SPECT/CT in a patient with parkinsonism. A 63-year-old man with parkinsonian symptoms for the past 12 months, initially diagnosed as Parkinson’s disease but with no appreciable response to L-DOPA treatment, was referred to brain scintigraphy. Transaxial SPECT and SPECT/CT (centre and bottom row) show reduced tracer uptake in the left putamen. CT (top row) demonstrates ipsilateral cortical atrophy. These combined findings suggest a diagnosis of corticobasal atrophy with associated parkinsonism, rather than typical Parkinson’s disease. *[Images provided courtesy of Dr. Duccio Volterrani, Regional Center of Nuclear Medicine, University of Pisa, Italy]*

SPECT imaging of brain perfusion is performed in cases of dementia and epilepsy using ^99m^Tc-HMPAO or ^99m^Tc-ethyl cysteinate dimer (ECD). The addition of CT to SPECT has not led to a breakthrough in this setting, in part because CT is not the procedure of choice for brain imaging, with MRI being the better tool. In the majority of cases, the diagnostic gain with SPECT/CT is only negligible [223–233]. Nonetheless, when comparison to age-matched healthy controls is required, such as the examination of the dopamine transporters with ^123^I-FP CIT in cases of parkinsonism (Fig. 11), CT information helps to obtain a more robust spatial normalization of SPECT data [232–234]. The use of SPECT/CT in brain tumours with tracers such as ^99m^Tc-MIBI and ^99m^Tc-tetrofosmin (Myoview^®^) or ^99m^Tc-bis-methionine-DTPA (MDM) is limited and largely restricted to differentiation between recurrence and radiation necrosis [235].

### Gastrointestinal tract

Active gastrointestinal (GI) bleeding is detected by contrast angiography or endoscopy if present at the time the study is performed. ^99m^Tc-labelled red blood cell (RBC) scintigraphy is a highly sensitive, non-invasive tool to detect intermittent bleeding. Positive studies identify patients who need immediate treatment if the site of bleeding is localized [236]. SPECT/CT was found to improve bleeding source localization in over 30% of cases [237] and, increased sensitivity from 89% to 93% compared to planar scans, with improved positional accuracy of the bleeding site in 92% vs. 74% of patients [238]. Timing of ^99m^Tc-RBC SPECT/CT acquisition needs further evaluation. There is also concern that during the relatively long SPECT/CT acquisition, patient motion or bowel artefacts can affect correct localization of the bleeding site [239, 240].

## Paediatrics applications

The use of SPECT/CT in children and adolescent patients can reduce the number of equivocal studies and spare further diagnostic workup [241]. However, the practice of CT in children requires expertise to balance the best diagnostic yield with minimum radiation burden [242, 243]. The use and extent of the CT field should be selected based on clinical question, scintigraphic findings, and previous imaging modalities.

**Fig. 12:**
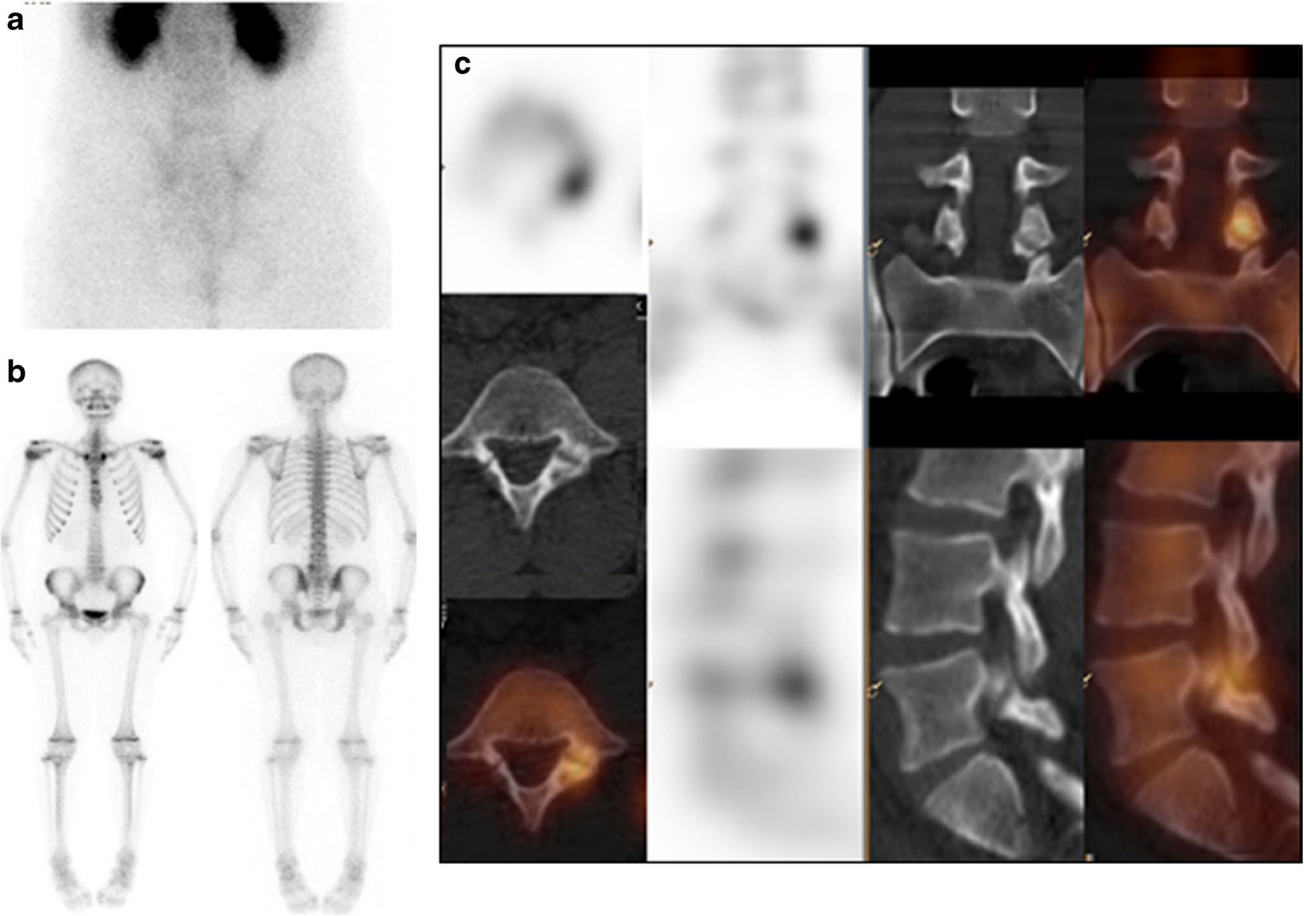
Bone SPECT/CT diagnosis of spondylolysis in an adolescent patient. A14-year-old girl was referred for evaluation of persistent low back pain. Early planar scan of lumbar spine and pelvis (**a**) shows no hyperaemia. Delayed WB-BS, anterior and posterior views (**b**) show a focus of abnormal ^99m^Tc-MDP uptake in left aspect of L5 vertebra, localized by SPECT/CT (**c**) to the left L-5 articular facet, consistent with spondylolysis

Bone SPECT/CT in children is performed mainly in benign conditions [244, 245]. Although MRI has taken over the main imaging role, in centers with no easy access to it, BS with SPECT/CT is a helpful option [246]. In orthopaedics, sports, and traumatic injuries in children, SPECT/CT can identify the pain generator at the cortical bone level, where MRI is less sensitive. In adolescent back pain, including spondylolysis, SPECT/CT is of value when X-rays and MRI have failed to identify the source of pain (Fig. 12) [247]. BS with targeted SPECT/CT plays a role in children with suspected bone and joint infections but without localizing symptoms, such as OM, spondylodiscitis, and septic arthritis [244, 245, 248]. Additional clinical indications for SPECT/CT in children include radiologically occult stress fractures and evaluation of congenital skeletal abnormalities of the spine or the extremities [249].

**Fig. 13:**
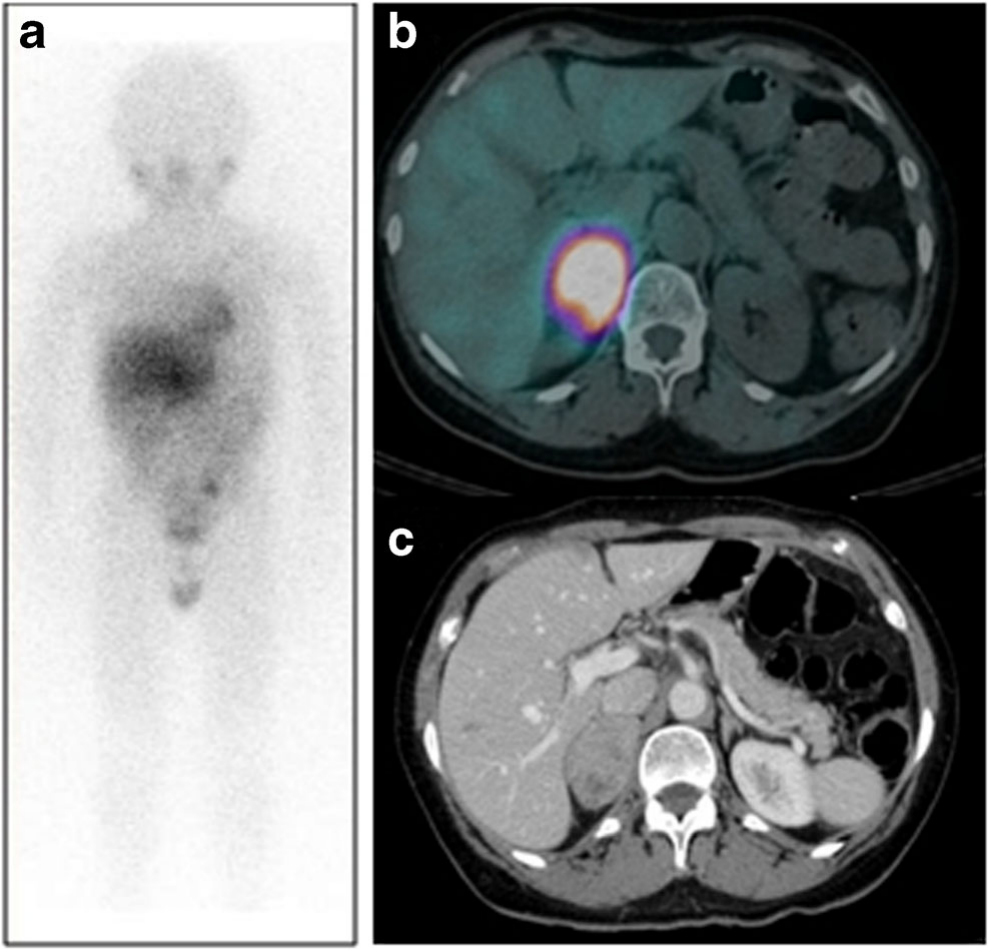
^123^I-mIBG SPECT/CT in neuroblastoma. A 6-year-old child with newly diagnosed neuroblastoma was referred for staging. Planar WB scan (**a**) shows an area of increased uptake in the right upper abdomen, partially overlapping the liver of unclear location and clinical significance. SPECT/CT (**b**) localizes this abnormal tracer uptake to the primary lesion in a large mass in the right adrenal seen on CT (**C**)

In paediatric solid tumours, SPECT/CT is used mainly with neuroblastic tumours and thyroid cancer. Neuroblastoma occurs in the adrenals and sympathetic ganglia. CeCT or MRI evaluate the size and position of the primary tumour in relationship to surrounding organs. ^123^I-mIBG SPECT/CT improves diagnostic accuracy (Fig. 13) and reduces the incidence of equivocal planar findings [250]. Sedation or general anaesthesia is often required for this lengthy examination in young children. Acquisition of a fully diagnostic ceCT as part of the SPECT/CT study is an option, in order to avoid an additional examination, thus providing a “one-stop shop” evaluation with a single exposure to anaesthesia [250, 251].

DTC, although uncommon, has a rising incidence in children who tend to have a more aggressive type as compared to adults [252]. ^123^I-iodide WBS with SPECT/CT is not recommended in low-risk disease but provided important information on residual thyroid tissue, nodal and distant metastases in patients with high risk DTC [253, 254].

Meckel diverticulum is the most frequent cause of lower GI haemorrhage in children. SPECT/CT improved diagnosis and localization of ectopic gastric mucosa, mainly in uncommon sites, and identified artefactual foci [242, 255]. Limited literature evidence indicates that SPECT/CT can potentially increase the diagnostic yield of ^99m^Tc-WBC imaging in paediatric infections [256] and of MPI in assessing congenital heart disease, complementary to echocardiography [257].

## Incidental CT findings

Because of the rapid increase in the use of diagnostic-quality SPECT/CT studies, incidentalomas, i.e. unexpected findings not related to the initial diagnostic inquiry, are often detected on the CT component. The reporting physicians should be aware of these incidental findings and their clinical significance. Incidentalomas have been categorized as major, moderate, or minor findings (Online Table 13). In the presence of major incidental findings, the referring clinician should be informed, and further investigations are in order to prevent adverse health effects. Moderate findings usually require further investigation, but their clinical impact is unclear. Minor findings rarely require further investigation and are unlikely to have adverse health effects [258–265].

## Concluding remarks

Within the domain of radionuclide-based diagnostic procedures, hybrid imaging has gained wide popularity, largely following the introduction of PET/CT into clinical routine. The metabolic or functional information provided by PET or SPECT is enhanced not only because of better AC, but also, and most importantly, due to the correlative assessment of altered tracer distribution with anatomical structures. This results in better performance indices for the diagnostic imaging procedure. Through hybrid imaging in general, and SPECT/CT in particular, the nuclear medicine expert brings an important contribution to better healthcare by tailoring clinical interventions to the individual patient’s needs. The authors are aware that “competitive” PET/CT methods exist for many of the indications discussed in the present review. When available, these methods are mentioned (see also online Tables). However, an extensive review of these other modalities was not the focus of this article.

By analysing the most relevant of over 400 papers published so far on the topic of clinical SPECT/CT, the current review provides an update on the established evidence. It demonstrates the definite advantages of SPECT/CT over planar and/or stand-alone SPECT in a variety of diseases. For most of the applications described above, SPECT/CT is already fully integrated into the routine clinical decision-making process. The information gathered whilst performing this review also points to areas where the application of integrated SPECT/CT imaging has not yet been proven to have definite advantages. The lack of large-scale studies and clear evidence-based proof do not permit the authors at present to translate the implementation of SPECT/CT into significant clinical impact for management. The potential of SPECT/CT imaging for lesion characterization will increase, especially following the use of diagnostic ceCT. Recent technological advances will also enhance the role to be played by quantitative SPECT/CT for dosimetry estimates in theragnostics, a topic that deserves a separate, dedicate assessment of current knowledge and contributions. It is therefore expected that the literature and conclusions summarized above will undergo continuing significant changes. The present data and trends for the near future reinforce, with respect to SPECT/CT, the axiom stated by the Greek philosopher Aristotle (384–322 BC) that “the whole is greater than the sum of its parts”.

## Electronic supplementary material

ESM 1 (PDF 845 kb)
