# Supplementary material for: Two decades of SPECT/CT – the coming of age of a technology: An updated review of literature evidence
Source: Eur J Nucl Med Mol Imaging. 2019 Jul 4;46(10):1990–2012. doi: 10.1007/s00259-019-04404-6 (PMC6667427; doi:10.1007/s00259-019-04404-6)
Supplement: Supplementary file 1 — (PDF 845 kb) [file 259_2019_4404_MOESM1_ESM.pdf]

Table 1: Literature evidenced on the role of SPECT/CT and SLN assessment in breast cancer.

Author/Source/Year<br>No. patients (n)<br>Type of study	Aims & Results
Giżewska, Nucl Med Commun. 2017 (1)<br>n=153<br>Retrospective	*Comparison with planar*<br>Detection rates: SPECT/CT: 77.7% (first echelon)<br>34.6% (second echelon LN)
Borrelli et al, Eur J Nucl Med Mol Imaging. 2017 (2)<br>n=122<br>Retrospective	*Comparison with planar*<br>Detection rates:<br>SPECT/CT: 53.3% vs. planar: 43.4%<br>Change in surgical approach: 21.3 % patients.
Zetterlund et al, Breast. 2016 (3)<br>n=37<br>Prospective	SPECT/CT detection rates: 91.9% patients
Pouw et al, Eur J Surg Oncol. 2016 (4)<br>n=284<br>Retrospective	SPECT/CT detection rates: 23.2% patients
Jimenez-Heffernan et al, J Nucl Med. 2015 (5)<br>n=1,182<br>Retrospective	*Comparison with planar*<br>Detection rate: SPECT/CT >planar<br>Drainage basin mismatch: 16.5%<br>Change in surgical approach: 17% patients.
Tomiguchi et al, Surg Today. 2016 (6)<br>n=381<br>Retrospective	*Comparison with planar*<br>SPECT/CT higher detection rate
Shima et al, Exp Ther Med. 2014 (7)<br>n=92<br>Retrospective	SPECT/CT detects level II/III LNs that may be at risk of metastatic involvement
Kraft et al, Nucl Med Rev Cent East Eur. 2013 (8)<br>n=320<br>Retrospective	*Comparison with planar*<br>SPECT/CT: higher detection rate<br>SPECT/CT: precise localization of all visualized SLNs
Yoneyama et al, Clin Nucl Med. 2014 (9)<br>n=106<br>Prospective	*Comparison with planar*<br>Detection rates: SPECT/CT: 100% vs. planar: 97.2%
Brouwer et al, Eur J Nucl Med Mol Imaging. 2012 (10)<br>n=50<br>Prospective	*Comparison with planar*<br>SPECT/CT detected significantly more SLNs (axillary, mammary, interpectoral)
Uren et al, Breast. 2012 (11)<br>n=741<br>Retrospective	SPECT/CT detection of SLNs: in 1 single node field - 63%, in 2 fields - 36%, and only few in 3 & 4 fields

Coffey et al, Nucl Med Commun. 2010 (12)<br>n=187<br>Retrospective	*Comparison with planar*<br>SPECT/CT : higher detection rate<br>SPECT/CT: precise localization of all visualized SLNs
Cheville et al, Breast Cancer Res Treat. 2009 (13)<br>n=32<br>Prospective	*Comparison with planar*<br>SPECT/CT characterized incidental findings & directed therapy to reduce long-term morbidity
vanDer Ploeg et al, Eur J Nucl Med Mol Imag. 2009 (14)<br>n=15<br>Prospective	*Comparison with planar*<br>SPECT/CT: detected lymphatic drainage in 53% additional patients<br>SPECT/CT: detected axillary SLN in 15% patients with known extra-axillary SLNs on planar
Gallowitsch et al, Nuklearmedizin. 2007 (15)<br>n=51<br>Prospective	*Comparison with planar*<br>SPECT/CT: more accurate characterization of SLNs (size, depth, location).
Lerman et al, J Nucl Med. 2007 (16)<br>n=220<br>Retrospective	*Comparison with planar; obese patients*<br>Detection rates:<br>Total population: SPCT/CT 91% vs. planar: 78%<br>Obese patients: SPECT/CT: 89% vs. planar: 72%

LN - lymph node; SLN – sentinel lymph node

Table 2: Literature evidence on the role SPECT/CT in SLN assessment in melanoma.

**Author/Source/Year**<br>**No. patients (n)**<br>**Type of study**	**Aims & Results**
Trinh et al, Ann Surg Oncol. 2018 (17)<br>n=118<br>Retrospective	**Melanoma in Head and Neck**<br>*Comparison with planar*<br>Detection rates: SPECT/CT: 100% vs. planar: 61.9% .<br>Change in surgical approach: 81% patients
Doepker et al, Ann Surg Oncol. 2017 (18)<br>n=351<br>Retrospective	*Comparison with planar*<br>Detection rates: SPECT/CT: 89.6% vs. planar: 50.4%
Jimenez-Heffernan et al, J Nucl Med. 2015 (5)<br>n=262<br>Retrospective	*Comparison with planar*<br>Detection rates: SPECT/CT > planar<br>Drainage basin mismatch: 11.1% patients<br>Change in surgical approach: 37% patients
Stoffels et al, Eur J Nucl Med Mol Imaging. 2014 (19)<br>n=464<br>Retrospective	*Comparison with planar*<br>Median cost of SLN procedure: SPECT/CT: 1,619.7 € vs. planar: 2,330.2 €; Cost savings by SPECT/CT 30.5 %
Zender et al, Am J Otolaryngol. 2014 (20)<br>n=14<br>Retrospective	SPECT/CT change in management: 57% patients.
Kraft et al, Nucl Med Rev Cent East Eur. 2013 (8)<br>n=161<br>Retrospective	*Comparison with planar*<br>SPECT/CT: significantly higher detection rate<br>SPECT/CT: precise localization of all visualized SLNs.
Fairbairn et al, J Plast Reconstr Aesthet Surg. 2013 (21)<br>n=32<br>Retrospective	*Comparison with planar*<br>Similar diagnostic accuracy<br>Change in surgical approach: > 30% patients
Vuthaluru et al, Am J Ophthalmol. 2013 (22)<br>n=12<br>Prospective	*Melanoma in eyelid*<br>SPECT/CT accurately localization of SLNs: 11/12 patients
Stoffels et al, JAMA. 2012 (23)<br>n=464<br>Retrospective	*Comparison with planar*<br>Detection rates: SPECT/CT > planar<br>Local relapse rate: SPECT/CT cohort 6.8% vs. standard cohort 23.8%<br>4-year DFS: SPECT/CT 93.9% vs planar 79.2%
Kraft et al, Nucl Med Rev Cent East Eur. 2012 (24)<br>n=113<br>Retrospective	*Comparison with planar*<br>Detection rates: SPECT/CT: 94.7% vs. planar: 88.5%

Veenstra et al, Ann Surg Oncol. 2012 (25)<br>n=631<br>Retrospective	*Melanoma in upper limb or trunk*<br>SPECT/CT detected lymphatic drainage to cervical basins, mainly IV and Vb.
Veenstra et al, Ann Surg Oncol. 2012 (26)<br>n=35<br>Prospective	*Comparison with planar*<br>Similar detection rates<br>SPECT/CT: localization & change in surgical approach: >30% patients
Nielsen et al, Eur J Nucl Med Mol Imaging. 2011 (27)<br>n=307<br>Retrospective	*Comparison with planar*<br>SPECT/CT detection rate: additional 10% patients
Klode et al, J Eur Acad Dermatol Venereol 2011 (28)<br>n=48<br>Retrospective	*Melanoma in Head & Neck*<br>*Comparison with planar*<br>SPECT/CT: better postoperative aesthetic results, lower morbidity; significantly shorter operating time with subsequent reduced costs
Vermeeren et al, Head Neck. 2011 (29)<br>n=38<br>Retrospective	*Melanoma in Head & Neck*<br>SPECT/CT detection rate: additional LNs in 16% patients<br>Change in surgical approach: >35% patients
van der Ploeg et al, Ann Surg Oncol. 2009 (30)<br>n=50<br>Retrospective	Most (involved) SLNs located in infero-medial & central zones<br>High frequency of pelvic second-tier nodes - need for deep groin dissection in most patients with positive SLNs.
van der Ploeg, Ann Surg Oncol. 2009 (31)<br>n=85<br>Retrospective	*Comparison with planar*<br>Detection rates: SPECT/CT > planar<br>Change in surgical approach in 35% patients; questionable value in 22% & no value in 42% patients
Ishihara et al, Int J Clin Oncol. 2006 (32)<br>n=35<br>Retrospective	*Comparison with blue dye*<br>SPECT/CT: higher detection rates in neck area
Even-Sapir et al, J Nucl Med. 2003 (33)<br>n=34<br>Prospective	*Comparison with planar*<br>SPECT/CT: higher detection rates

SLN – sentinel lymph node
DFS – disease free survival

Table 3: Literature evidence on the role of pre-ablation diagnostic SPECT/CT in differentiated thyroid cancer

**Author/Source/Year**<br>**No. patients (9)**<br>**Type of study**	**Results**
Avram et al, J Clin Endocr Metab, 2015 (34)<br>n=220<br>Prospective	SPECT/CT (combined with stimulated serum thyroglobulin)<br>• Change in risk stratification: 15% patients<br>• Change in intended management: 31% patients
Agrawal K et al, Ind J Nucl Med, 2015 (35)<br>n=83<br>Prospective	SPECT/CT:<br>• Detection of additional metastases: cervical nodes 29% & distant location 10% patients<br>• Change in TNM stage: 9.6% patients<br>• Change in risk stratification: 13.2% patients<br>• Change in intended management: 38.5% patients
Avram et al, J Clin Endocr Metab, 2013 (36)<br>n=320<br>Prospective	SPECT/CT:<br>• Detection of additional cervical metastases: 38% patients under 45y & 24% patients over 45y of age<br>• Detection of additional distant metsastases: 4% patients under 45y & 10% patients over 45y of age
Wong et al, Am J Roentgenol, 2010 (37)<br>n=48<br>Retrospective	SPECT/CT:<br>• Additional findings: 40% patients<br>• Change in post-surgical stage: 21% patients<br>• Change in intended management (RAI therapy dose):58% patients

RAI – radioactive Iodine

Table 4: Literature evidence on the role of SPECT/CT after radioiodine treatment for ablation or for recurrent/metastatic differentiated thyroid cancer

**Author/Source/Year**<br>**No. patients (n)**<br>**Type of study**	**Results**
Hassan et al, Europ Thyroid, 2015 (38)<br>n=67 (ablation 29; therapy 38)<br>Retrospective	SPECT/CT:<br>• Reduced number of equivocal foci from 17 to 1<br>• Change in stage: 20.8% patients<br>• Change in management: 14% patients
Grewal et al, J Nucl Med, 2010 (39)<br>n=148 (ablation 109; therapy 39)<br>Retrospective	SPECT/CT:<br>• reduced number of equivocal foci by 70%<br>• spared further CT/MR: 6.6% patients<br>• change in risk stratification: 6.4% patients post-ablation<br>• similar performance post-ablation & post-therapy.
Kohlfuerst et al, Eur J Nucl Med Mol Imag, 2009 (40)<br>n=53 (ablation 23; therapy 18)<br>Prospective	SPECT/CT:<br>• Unexpected lesions: 28.9%<br>• Overall diagnostic impact: 63.6% patients<br>• Change in N status: 36.4% patients<br>• Change in M status: 21.1% patients<br>• Change in management: 24.4% patients
Spanu et al, J Nucl Med, 2009 (41)<br>n=117 (ablation 108; therapy 9)<br>Prospective	SPECT/CT:<br>• Overall diagnostic impact: 67.8% patients<br>• Change in management: 35.6% patients<br>• Sparing unnecessary imaging/treatment: 20.3% patients.
Wang et al, Clin Imaging, 2009 (42)<br>n=94 (not specified)	SPECT/CT:<br>• Better localization of uptake: 21% patients<br>• Overall diagnostic impact: 12.8% patients<br>• Change in management: 23.4% patients
Wong et al, Am J Roentgenol, 2008 (43)<br>n=56 (ablation 52; therapy 4)<br>Retrospective	SPECT/CT:<br>• diagnostic impact: cervical nodes 40.8% & distant foci 100%<br>• increase in diagnostic confidence in 70.7% lesions
Tharp et al, Eur J Nucl Med Mol Imag, 2004 (44)<br>n=71 (not specified)<br>Retrospective	SPECT/CT:<br>• diagnostic value: 57% patients (including 27% patients with equivocal cervical uptake & 13% patients with distant foci)

Table 5: Literature evidence on the role of SPECT/CT in Neuroendocrine Neoplasms

**Author/Source/Year**<br>**No. patients (n)**<br>**Tracer & Type of tumor**<br>**Type of stud,**	**Aims & Results**
Kunikowska et al, Clin Nucl Med 2017 (45)<br>n=68<br>99mTc-HYNICTOC, NET<br>Retrospective	*Comparison with Ga-DOTATATE PET/CT:*<br>SPECT/CT : sensitivity: 82% , specificity 69 %, PPV 92%, NPV 47%, accuracy 79%<br>PET/CT: sensitivity 100%, specificity: 85%, PPV: 97%, NPV: 100%, accuracy: 97%<br>Detection rate: SPECT/CT < PET/CT
Trogrlic et al, Nuklearmedizin. 2017 (46)<br>n=65<br>99mTc-HYNICTOC, NET<br>Retrospective,	*Comparison with planar+ SPECT:*<br>SPECT/CT: Sensitivity 88.9, specificity 79.3,<br>Accuracy: SPECT/CT 88.9% vs SPECT 73.8%<br>SPECT/CT: change in management 16.9%
Etchebehere et al, J Nucl Med. 2014 (47)<br>n=19<br>99mTc-HYNICTOC, GEP<br>Prospective	*Comparison Ga-DOTATATE PET/CT & MR:*<br>Sensitivity: 60% SPECT/CT, 96% PET/CT, 72% MR<br>Specificity: 99% SPECT/CT, 97% PET/CT, 100% MR<br>PPV: 96% SPECT/CT, 94% PET/CT, 100% MR<br>NPV: 83% SPECT/CT, 98% PET/CT, 88% MR<br>Accuracy: 86% SPECT/CT, 97% PET/CT, 91% MR
Spanu et al, Am J Nucl Med Mol Imaging. 2017 (48)<br>n=104<br>111In-pentetreotide, GEP<br>Retrospective	*Comparison with CI:*<br>Sensitivity: 91.4% SPECT/CT vs 71.4% CI<br>Accuracy: 94.2% SPECT/CT vs 80.8% CI<br>Change in management: 27.9% SPECT/CT vs 9.6% CI
Ait et al, Nucl Med Commun. 2017 (49)<br>n=13<br>111In-pentetreotide, Tumour of pancreas<br>Retrospective	Comparison with planar:<br>SPECT/CT: better localization & quantification
Ruf et al, J Nucl Med. 2016 (50)<br>n=31<br>111In-pentetreotide, GEP<br>Prospective	*Diagnostic SPECT/CT; Comparison with CT:*<br>Detection rate: dSPECT/CT 78-89% vs. CT 63-85%<br>dSPECT/CT change in management 25.8% patients
Lee et al, Nucl Med Mol Imaging. 2015 (51)<br>n=13<br>111In-pentetreotide, NET<br>Prospective	*Comparison with Ga-DOTATATE PET/CT:*<br>Sensitivity: 54% SPECT/CT vs. 100% PET/CT

Chiaravalloti et al, Anticancer Res. 2015 (52)<br>n=81<br>111In-pentetreotide , Lung carcinoid<br>Prospective	*Comparison with ceCT:*<br>Primary/local recurrence<br>Sensitivity 96% SPECT/CT vs. 87.5% CT<br>Specificity 92% SPECT/CT vs. 97% CT<br>Distant mets:<br>Sensitivity 85.5% SPECT/CT vs. 75.2% CT<br>Specificity 84.6% SPECT/CT vs. 90.5% CT
Sainz-Esteban et al, Nucl Med Commun. 2015 (53)<br>n=107<br>111In-pentetreotide, NET<br>Retrospective	*Comparison with planar*:<br>Detection rate: 94.4% SPECT/CT vs 65.6% planar<br>SPECT/CT: 87.8% sensitivity,96.6% specificity<br>Change management in 11% patients
Schreiter et al, Radiol Oncol. 2014 (54)<br>n=123<br>111In-pentetreotide, NET<br>Retrospective	*Comparison with Ga-DOTATATE PET/CT:*<br>PET/CT is better than SPECT/CT
Wong et al, Acad Radiol. 2010 (43)<br>n=49<br>111In-pentetreotide, GEP<br>Prospective	*Comparison with planar:*<br>SPECT/CT improved lesion localization: 61.8%<br>SPECT/CT changed lesion classification: 28.1%<br>SPECT/CT diagnostic value: 28.6%
Apostolova et al , Ann Nucl Med. 2010 (55)<br>n=25<br>111In-pentetreotide, NET<br>Prospective	Comparison with planar:<br>up-staging 18% lesions & down-staging 12% lesions
Castaldi et al, Radiol Med. 2008 (56)<br>n=54<br>111In-pentetreotide, NET<br>Retrospective	*Comparison with planar:*<br>Change in management: 26% patients
Perri et al, Q J Nucl Med Mol Imaging. 2008 (57)<br>n=81<br>111In-pentetreotide, NET<br>Retrospective	*Comparison with SPECT:*<br>Detection rate:<br>Patient analysis: 92.6% SPECT/CT vs. 79% SPECT<br>Lesion analysis 96.4% SPECT/CT vs. 81.1% SPECT
Hillel et al, Clin Radiol. 2006 (58)<br>n=29<br>111In-pentetreotide, NET Prospective	Comparison with planar:<br>Change in management: 64% patients
Krausz et al, Clin Endocrinol 2003 (59)<br>n=71<br>111In-pentetreotide, NET (n=67) & MTC (n=4)<br>Retrospective	*Comparison with planar:*<br>Change in diagnosis: 32% patients<br>Change in management: 14% patients
Chang et al, Cancer Imaging. 2016 (60)<br>n=23<br>123I-mIBG, Pheochromocytoma	*Comparison with Ga-DOTATATE PET/CT:*<br>Similar performance

Prospective	
Kroiss et al, Ann Nucl Med. 2017 (61)<br>(n=10)<br>123I-mIBG, Pheochromocytoma<br>Prospective	*Comparison with 18F-DOPA PET/CT:*<br>Detection rates: 20.0% SPECT/CT vs. 100% PET/CT<br>Sensitivity: 11.1% SPECT/CT vs. 69.2% PET/CT
Nakamoto et al, Clin Nucl Med. 2016 (62)<br>(n=68)<br>123I-mIBG, Pheochromocytoma<br>Prospective	SPECT/CT quantification
Kroiss, Eur J Nucl Med Mol Imaging. 2015 (63)<br>(10)<br>123I-mIBG, Pheochromocytoma<br>Prospective	*Comparison with Ga-DOTATATE PET/CT:*<br>Detection rate: 20.0% SPECT/CT vs.100% PET/CT<br>Sensitivity: 6.9% SPECT/CT vs. 100% PET/CT
Derlin et al, Clin Nucl Med. 2013 (64)<br>(n=22)<br>123I-mIBG, Pheochromocytoma<br>Prospective	*Comparison with MRI:*<br>SPECT/CT: sensitivity 87.5%, specificity 93.8%, accuracy 92.5%<br>MRI: sensitivity 87.5%, specificity 96.9%, accuracy 95%.<br>SPECT/MRI fusion superior to both SPECT/CT and MRI (sensitivity 100%)
Fukuoka et al, Clin Nucl Med. 2011 (65)<br>(n=16)<br>123I-mIBG and 131I-mIBG (post-therapy), Pheochromocytoma<br>Prospective	*Comparison with planar:*<br>Detection rate – lesion-based analysis:<br>-123I-mIBG SPECT/CT: additional 20%<br>-131I-mIBG SPECT/CT: additional 5%<br>Additional diagnostic information:<br>-123I-mIBG SPECT/CT: 81% studies<br>-131I-mIBG SPECT/CT: 53% studies
Meyer-Rochow et al, Ann Surg Oncol. 2010 (66)<br>(n=22)<br>123I-mIBG, Pheochromocytoma<br>Prospective	Additional information from SPECT+CT (correlative imaging) : 6 patients

NET – neuroendocrine tumour; GEP – Gastro-entero-pancreatic tumour;
PPV – positive predictive value; NPV – negative predictive value
CI – conventional imaging; CeCT – contrast enhanced CT

Table 6: Literature evidence on the performance indices of bone SPECT/CT in cancer patients.

**Author/Source/Year**<br>**No. patients (n)**<br>**Gold standard**	**Sensitivity (%)**	**Specificity (%)**	**Significance**<br>**(p < 0.05)**
Zhao et al, Skel Radiol, 2010. (67)<br>n=125<br>Biopsy & radiological follow-up	SPECT 82.5<br>SPECT/CT 66.7	SPECT 66.7<br>SPECT/CT 98.4	Specificity & accuracy
Palmedo et al, Eur J Nucl Med Mol Imag, 2014. (68)<br>n= 308<br>Clinical follow-up	WBS 93<br>SPECT 94<br>SPECT/CT 97	WBS 78<br>SPECT 71<br>SPECT/CT 94	Specificity
Zhang et al, Nuklearmedizin, 2015 (69)<br>n= 65<br>Pathology & clinical follow-up	SPECT 70.9<br>SPECT/CT 100	SPECT 94.9<br>SPECT/CT 97.4	NA
Haraldsen et al, Clin Physiol Funct Imag, 2016. (70)<br>n=73<br>MRI	WBS 87<br>SPECT 87<br>SPECT/ ldCT 79<br>SPECT/ dCT 84	WBS 63<br>SPECT 71<br>SPECT/ ldCT 63<br>SPECT/ dCT 83	Specificity
Jambor et al, Acta Oncol 2016. (71)<br>n= 53<br>consensus reading & clinical and imaging follow-up	WBS 62<br>SPECT 74<br>SPECT/CT 85<br>F-18-PET 93<br>wbMRI+DWI 91	WBS 50<br>SPECT 44<br>SPECT/CT 5<br>F-18-PET 6<br>wbMRI+DWI 4	Accuary of F-18-PET & wbMRI+DWI > WBS, SPECT & SPECT/CT
Fonager et al, Am J Nucl Med Mol Imag, 2017. (72)<br>n= 37<br>Clinical & imaging follow-up	WBS 78<br>SPECT/CT 89<br>F-18-PET/CT 89	WBS 90<br>SPECT/CT 100<br>F-18-PET/CT 90	NS
Mahaletchumy et al, World J Nucl Med, 2017. (73)<br>n= 85<br>Correlative imaging & clinical follow-up	WBS 43<br>SPECT 58<br>SPECT/CT 78	WBS 85<br>SPECT 92<br>SPECT/CT 94	NA

WBS – whole body planar bone scan
NA – not available
DWI – diffusion weighed imaging

Table 7: Literature evidence on the role of SPECT/CT for planning or assessment of trans-arterial radioembolization (TARE)

**Author/Source/Year**<br>**No. patients (n)**<br>**Type of study**	**Aims & Results**
Dittmann et al, J Nucl Med. 2018 (74)<br>n=50<br>Prospective	*Comparison with planar*.<br>SPECT/CT: significantly lower hepatopulmonary shunts, substantial shunting in 4% cases (vs. 20% for planar).
Yue et al, Med Phys. 2016 (75)<br>n=15<br>Prospective	*Comparison with PET/CT.*<br>Congruent results of 90Y Bremsstrahlung SPECT/CT and 90Y PET/CT in all cases
Erxleben et al, Acta Radiol. 2016 (76)<br>n=316<br>Retrospective	*Comparison with planar.*<br>SPECT/CT: significantly lower hepatopulmonary shunts
Theysohn et al, PLoS One. 2015 (77)<br>n=852<br>Retrospective	*Comparison with planar.*<br>SPECT/CT: unexpected extrahepatic uptake: 6.5% patients
Gates et al, J Nucl Med. 2015 (78)<br>n=174<br>Retrospective	*Comparison with planar.*<br>SPECT/CT: additional shunts identification
Ilhan et al, J Nucl Med. 2015 (79)<br>n=502<br>Retrospective	Wide variation of uptake among liver metastases subtypes
Spreafico et al, Cardiovasc Intervent Radiol. 2015 (80)<br>n=100<br>Retrospective	*Comparison with planar.*<br>SPECT/CT: identifies accessory branches in 19 lesions/17 patients, thus changing the embolization procedure
van den Hoven et al, Cardiovasc Intervent Radiol. 2014 (81)<br>n=110<br>Retrospective	SPECT/CT identified aberrant hepatic arteries: 34% patients
Zade et al, Nucl Med Commun. 2013 (82)<br>n=35<br>Prospective	*Comparison with PET/CT.*<br>Congruent results of 90Y Bremsstrahlung SPECT/CT and ^{90}Y PET/CT: 97.14% cases
Padia et al, J Vasc Interv Radiol. 2013 (83)<br>n=13<br>Prospective	*Comparison with PET/CT.*<br>^{90}Y PET/CT: higher spatial resolution & lower scatter

Burgmans et al, Eur J Radiol. 2012 (84)<br>n=79<br>Retrospective	*Comparison with planar.*<br>Detection rate of hepatic falciform artery: SPECT/CT: 13.3% vs. digital subtraction angiography: 11.9% vs. CT arteriography: 52.3%
Ahmadzadehfar et al, Eur J Nucl Med Mol Imaging. 2012 (85)<br>n=188<br>Retrospective	*Comparison with planar.*<br>Prediction of GI ulcers: SPECT/CT sensitivity 87%, specificity 100%, PPV 100%, NPV 99%, accuracy 99%
Lauenstein et al, Rofo. 2011 (86)<br>n=27<br>Prospective	*Comparison with planar.*<br>SPECT/CT only detected perfusion of occluded liver segment: 59% patients
Hamami et al, J Nucl Med. 2009 (87)<br>n=58<br>Prospective	*Comparison with planar+SPECT.*<br>Detection of GI shunting:<br>SPECT + CT fusion: sensitivity 100%, specificity 94%, accuracy 96%
Denecke et al, Eur Radiol. 2008 (88)<br>n=22<br>Prospective	*Comparison with SPECT.*<br>Detection of GI shunting: SPECT/CT 31% vs. SPECT 15% patients

GI - gastrointestinal
PPV – positive predictive value; NPV – negative predictive value

Table 8: **L**iterature evidence on the role of bone SPECT/CT in benign bone conditions.

**Author/Source/Year**<br>**No. patients (n)**<br>**Reason for study, Anatomical region**<br>**Type of study**	**Aim & Results**
Russo VM et al, World Neurosurg, 2017 (89)<br>n=99<br>LBP, SPECT/CT of spine<br>Prospective	*Compare SPECT/CT patterns with CT joint degeneration & Modic changes and MRI disc abnormalities*<br>SPECT/CT: localization of active facet joints, better LBP management<br>SPECT/CT patterns: no correlation with degree of CT degeneration >40%<br>SPECT/CT uptake: high agreement with Modic changes
Hudyana et al, Eur J Nucl Med Mol Imag, 2016 (90)<br>n=48<br>s/a lumbar arthroscopy with screw insertion, SPECT/CT of spine<br>Retrospective	*Accuracy for diagnosis of loosening of fixation material in back pain after surgery*<br>High sensitivity & specificity for exclusion of screw loosening<br>SPECT/CT identified other causes of recurrent LBP
Sumer J et al, Nucl Med Comm, 2013 (91)<br>n=37<br>s/a lumber fusión surgery; SPECT/CT of spine<br>Retrospective study	*Value in LBP after surgery, compared to planar+SPECT*<br>SPECT/CT: significantly higher accuracy; procedure of choice
Ha S et al 2015 (92)<br>n=50<br>Feet pain, SPECT/CT of feet & ankle<br>Retrospective	*Diagnostic performance with regard to lesion type, compared to MRI*<br>SPECT/CT & MRI: comparable diagnostic performance<br>SPECT/CT & MRI: complementary techniques
Chicklore S et al, Nucl Med Comm, 2013 (93)<br>n=209<br>Feet pain; SPECT/CT of feet & ankle<br>Retrospective	*Diagnostic accuracy for impingement syndrome & ST pathology; compared to MRI & US*<br>SPECT/CT similar performance
Huellner MW et al, PLoS One, 2013 (94)<br>n=32<br>Wrist pain; SPECT/CT of hands & wrists<br>Retrospective	*Diagnostic accuracy & interobserver agreement compared to MRI, CT, X-rays, planar BS*<br>SPECT/CT: most helpful modality<br>MRI: better characterization of lesion type<br>Good interobserver agreement (accuracy, localization, etiology)
Schleich FS et al, Eur J Nucl Med Res, 2012 (95)<br>n=51<br>Wrist pain; SPECT/CT of hands & wrists<br>Retrospective	*Diagnostic, therapeutic impact: compared to X-rays & planar BS*<br>SPECT/CT: highest lesion detectability; impact on patient management
Dobrindt O et al, BMC Med Imaging, 2015, (96)<br>n=50<br>Painful THR & TKR; SPECT/CT of hips or knees<br>Retrospective	*Compared to 3-phase BS + SPECT*<br>SPECT/CT: higher diagnostic accuracy in (a)septic loosening
Chew CG et al, Annals Nucl Med, 2010, (97)<br>n=117<br>Patello-femoral disorders; SPECT/CT of knees	*SPECT/CT arthrography for evaluation of mechanical loosening of prostheses.*

Retrospective	SPECT/CT of hip: better for acetabular cup but not for femoral stem.<br>SPECT/CT of knee: better in femoral and tibial component
Slevin O et al, 2017 (98)<br>n=104<br>Patellofemoral disorders; SPECT/CT of knees<br>Retrospective	*Tracer distribution patterns in patellar resurfacing*<br>SPECT/CT of value for evaluation of patello-femoral disorders after TKA

LBP – Low back pain
THR – Total hip replacement
TKR – Total knee replacement
TKA – Total knee arthroplasty
ST – soft tissues
BS – bone scan

Table 9: Literature evidence on the role of SPECT/CT in musculo-skeletal infections.

**Author/Source/Year**<br>**No. patients (n)**<br>**Tracer, Clinical indication**<br>**Type of study**	**Aims & Results**
Horger et al, EJNM 2003 (99) (n=27)<br>Post-traumatic OM, 99mTc-AGA	*Compared to BS::*<br>Specificity: SPECT/CT 89% vs. SPECT 78%<br>Same sensitivity 100%.<br>SPECT/CT: better diagnostic accuracy to differentiate OM from STI
Filippi et al, JNM 2006; (100)<br>n=28<br>OM/infected joint prosthesis, 99mTc-WBC<br>Prospective	SPECT/CT: accurate localization of all positive foci.<br>SPECT/CT: improved diagnosis 36% patients (ST vs. bone; complicated bone after trauma; synovial infection without prosthesis involvement)
Bar-Shalom et al, JNM 2006 (101)<br>n=32, Ga-67 (n=21); In-WBC (n=11)<br>Mixed population<br>Retrospective	*Compared to planar + SPECT:*<br>SPECT/CT: role in diagnosis, localization, extent of disease<br>SPECT/CT contribution: WBC > Ga
Horger et al, Arch Orth Surg 2007 (102)<br>n=31<br>Mixed population, 99mTc-HEDP<br>Prospective	*Compared to 3-phase planar + SPECT:*<br>Specificity SPECT/CT 86%; vs. BS 50%;<br>Same sensitivity 78%.<br>SPECT/CT avoids false positives & equivocal findings
Sathekge et al, Annals Nucl Med 2018 (103)<br>n=184<br>OM vs. STI, 99mTc-Ubi	*Compared to planar + SPECT:*<br>SPECT/CT sensitivity 99%, specificity 95%, PPV 93%, NPV 99%, accuracy 95%<br>SPECT/CT: improved diagnostic confidence in 49% patients; better interobserver agreement
Djekidel et al, Clin Nucl Med 2011 (104)<br>n=43<br>Mixed population, In111- & Tc99m-WBCs<br>Retrospective	*Compared to planar + SPECT:*<br>SPECT/CT: sensitivity 88%, specificity 85%, PPV 84%, NPV 89%.<br>Increase in correct lesion location.<br>Improved overall reader confidence.<br>No difference between 111In- & 99mTc-WBCs SPECT/CT<br>No difference before & after treatment
Filippi et al, JNM 2009 (105)<br>n=17<br>Diabetic Foot, 99mTc-WBC	Compared to 3-phase BS:<br>SPECT/CT change in interpretation: 53% patients<br>SPECT/CT did not contribute in negative scan
Heiba et al, J Foot Ankle Surg 2010 (106)<br>n=213<br>Diabetic foot, 99mTc-MDP & 111In WBC	DI - 2 steps: BS/WBCs-SPECT/CT ± WBCs/Bone marrow SPECT/CT<br>Diagnostic accuracy: DI > WBCs/BS; DI SPECT/CT > DI planar/SPECT only.<br>DI SPECT/CT: improves detection & discrimination of STI vs. OM

Erdman et al, Diab Care 2012 (107)<br>n=77<br>Diabetic foot, 99mTc-WBC<br>Retrospective	*SPECT/CT based CSI:*<br>Favorable outcome: CSI 0 = 92% ; CSI ≥7 = 25%<br>SPECT/CT: visual < CSI for predicting outcome
Aslangul et al, Diab Care 2013 (108)<br>n=55<br>Diabetic foot, Ga-67<br>Prospective	*Diagnosis of OMs & treatment tailoring; combined with biopsy:*<br>SPECT/CT + biopsy: sensitivity 88%; specificity 94%, PPV 92%, NPV 91%<br>SPECT/CT + biopsy: spared antibiotics 55% cases
Heiba et al, NM Comm 2013 (109)<br>n=227<br>Diabetic foot, 99mTc-MDP & 111In WBC<br>Retrospective	*Compared to CI in different population: SPECT/CT (n=232) & CI (n=227)*<br>DI SPECT/CT: more accurate diagnosis of OM, STI, other bony pathology<br>DI SPECT/CT: associated with shorter hospitalization length
Vouillarmet et al, Diab Med 2014 (110)<br>n=22<br>Diabetic foot, 99mTc-WBC<br>Retrospective	*Monitoring treatment response; compared to 3-phase BS & X-rays)*<br>Prediction of OM relapse after antibiotics:<br>SPECT/CT: sensitivity 100%, specificity 92%, PPV 72%, NPV 100%<br>Better than X-rays & BS<br>Negative WBC-SPECT/CT useful in guiding therapy.
La Fontaine et al, Wound 2016 (111)<br>n=110<br>Diabetic foot, 99mTc-WBC<br>Retrospective	*Compared to MRI in different population: SPECT/CT (n=52) & MRI (n=58)*<br>SPECT/CT: sensitivity 89%, specificity 35%, PPV 74%, NPV 60% vs. MRI: sensitivity 87%, specificity 37%, PPV 74%, NPV 58% (p NS)
Lazaga et al, Int Wound J 2016 (112)<br>n=20<br>Diabetic foot, 99mTc-WBC<br>Retrospective	*Monitoring response to treatment*<br>SPECT/CT: sensitivity 90%, specificity 56%, PPV 69%, NPV 83%.<br>Useful to determine treatment outcomes
Vouillarmet et al, Diabetologia 2017 (113)<br>n=45<br>Diabetic foot, 99mTc-WBC<br>Retrospective	*Monitoring response to treatment (at 6 & 12 weeks)*<br>SPECT/CT (12 weeks): sensitivity 100%, specificity 56%, PPV 46%, NPV 100%.<br>SPECT/CT predicted remission at end of treatment.
Fuster et al, Clin Nucl Med 2012 (114)<br>n=34<br>Spondylodiskitis, 67Ga<br>Prospective	*Compared to BS & FDG-PET/CT*<br>BS & Ga-67 SPECT/CT: sensitivity 78%, specificity 81%, PPV 82%, NPV 76%, accuracy 79%.<br>FDG-PET/CT better performance, concordance with SPECT/CT
Tamm et al, Can Assoc Radiol J (115)<br>n=34<br>Spondylodiskitis , 99mTc-MDP and/or 67Ga<br>Retrospective	*Compared to BS & MRI*<br>BS & Ga-67 SPECT/CT vs. MRI: same sensitivity (91%), specificity and PPV (100%); similar NPV (94% vs. 80%) and accuracy (97% vs 95%)
Lazzeri et al, Clin Nucl Med 2010 (116)<br>n=72<br>Spondylodiskitis, 111In-Biotin	*Early diagnosis; compared to planar & SPECT*<br>SPECT/CT vs. SPECT: similar sensitivity (94% vs. 92), same specificity (92%).

Prospective	SPECT/CT correctly localized infection to bone, ST or both: 22% cases
Chakraborty et al, Ind JNM 2013 (117)<br>n=20<br>OM of base of skull, 99mTc-MDP<br>Retrospective	*Compared to 3-phase BS*<br>SPECT/CT localized lesions to specific bone in 50% & showed destructive changes in 25%
Sharma et al, Jpn J Radiol 2013 (118)<br>n=13<br>OM of base of skull, 99mTc-MDP<br>Retrospective	*Compared to planar, SPECT & CT*<br>AUC for SPECT/CT 0.977 vs. SPECT 0.909, CT 0.886, planar 0.614<br>Accuracy SPECT/CT 92%, SPECT 85%, CT 77%, planar 46%
Bolouri et al, EJNMMI 2013, (119)<br>n=42<br>OM of jaw, 99mTc-MDP	*Compared to SPECT & orthopantomography (OPT)*<br>SPECT/CT sensitivity 100%, specificity 86%, accuracy 98 %<br>SPECT: 100%, 71%, 95%; CT 77%, 86%, 79%; OPT 59%, 100%, 66 %<br>SPECT/CT most useful but not cost justified
Graute et al, EJNMMI 2010 (120)<br>n=31<br>Low grade joint infection, 99mTc-AGA<br>Retrospective	*Compared to 3-phase planar*<br>SPECT/CT: sensitivity 89%, specificity 73%, PPV 57%, NPV 94%<br>SPECT/CT improvement in diagnosis, localization & extent
Kim et al, J Comput Assit Tomogr 2014 (121)<br>n=164<br>Infected hip & knee prostheses, 99mTc-WBC<br>Retrospective	*Compared to 2-phase BS*<br>SPECT/CT: sensitivity 93%, specificity 93%, PPV 94%, NPV 92%, accuracy 93%.<br>Higher impact of SPECT/CT on sensitivity & specificity for hip vs. knee prosthesis infections.

OM – osteomyelitis
ST – soft tissue
STI – ST infection
PPV – positive predictive value; NPV – negative predictive value
CI – conventional imaging
DI – dual isotope
CSI - Composite Severity Index CSI
OPT - orthopantomography

Table 10: Literature evidence on the role of SPECT/CT in soft tissue and visceral infections

**Author/Source/Year**<br>**No. patients (n)**<br>**Tracer, Clinical indication**<br>**Type of study**	**Aims & Results**
Bar-Shalom et al, J Nucl Med 2006 (101)<br>n=50<br>FUO; mixed STI (67Ga n=26); VGI (111In-WBC n=24)<br>Retrospective	*Compared to SPECT*<br>SPECT/CT: diagnosis & localization in 48% & extent in 43% patients<br>Excluded infection in 4 sites (67Ga bowel uptake)<br>Contribution: 111In-WBC > 67Ga
Lou et al, Nucl Med Comm 2010 (122)<br>n=11<br>99mTc-WBC; VGI<br>Retrospective	SPECT/CT: high accuracy in clinically suspected cases
Khaja, Clin Imag 2013 (123)<br>n=20<br>111In- WBC; VGI<br>Retrospective	*Compared to CTA & Software fusion*<br>Sensitivity, specificity, accuracy, PPV, NPV:<br>WBC: 75/100/80/100/50%<br>CTA: 88/50/80/88/50%<br>SPECT/CTA fusion: 94/50/85/88/67%<br>Software fusion: better diagnostic confidence; impact on outcome
Erba et al, Eur J Nucl Med Mol Imag 2014 (124)<br>n=55<br>99mTc-WBC; Late & low grade VGI<br>Prospective	*Compared to SPECT:*<br>SPECT/CT vs. SPECT: sensitivity 100% vs. 85%; specificity 100 % vs. 63%<br>SPECT/CT decreased FPs in 37% patients
Erba et al, J Nucl Med 2012 (125)<br>n=131<br>99mTc-WBC; IE<br>Prospective	Compared to echo<br>SPECT/CT sensitivity 90%, NPV 94%, specificity & PPV 100%<br>Main value in negative or difficult-to-interpret echo
Lauridsen et al, Int J Cadiovasc Imag 2017 (126)<br>n=55<br>99mTc-WBC; extracardiac sites of IE	*Compared to FDG*<br>Clinical utility score: FDG-PET/CT > WBC-SPECT/CT
Litzler et al, J Nucl Med 2010 (127)<br>n=13<br>99mTc-WBC; Infected CIED	*Monitoring response to antibiotic treatment*<br>SPECT/CT: extent & precise location of infection<br>SPECT/CT: better therapeutic strategies.
Erba et al, JACC 2013 (128)<br>n=63<br>99mTc-WBC; Infected CIED<br>Prospective	SPECT/CT confirmed diagnosis, defined extent & detected associated complications<br>SPECT/CT sensitivity: 94%; NPV 95%
Heiba et al, Nucl Med Comm 2017 (129)<br>(n=21)<br>Tc-MDP & In-WBC; Pelvic pressure sore	*Compared to planar*<br>DI- SPECT/CT: higher sensitivity, specificity & diagnostic confidence than either DI scintigraphy or SPECT/CT

Hung et al, Infect Dis 2017 (130)<br>n=58<br>67Ga; FUO	*Compared to FDG-PET/CT*<br>^{67}Ga-SPECT/CT: high FN rate (55%) vs. FDG-PET/CT high FP rate (44%)<br>Sensitivity: ^{67}Ga-SPECT/CT 79% vs. FDG-PET/CT 45%<br>Clinical contribution: ^{67}Ga-SPECT/CT 72% vs. FDG-PET/CT 55%
Nowosinska et al, World J Nucl Med (131)<br>n=18<br>67Ga; Infected kidneys in ESRF & renal transplant<br>Retrospective	SPECT/CT contributory: 80% kidneys in ESRF & 33% renal transplant patients.<br>SPECT/CT: 44% patients better location and/or extent; differentiating physiological from pathological uptake.

FUO – Fever of unknown origin
VGI – vascular graft infection
IE – infective endocarditis
CIED - cardiac implantable electronic devices
ESRF – end stage renal failure
CTA – CT angiography
FP –False positive; FN – False negative
DI – dual isotope

Table 11: Literature evidence on the role of SPECT/CT in primary hyperparathyroidism.

**Author/Source/Year**<br>**No. patients (n)**<br>**Type of study**	**Aim & Results**
Gayed et al, J Nucl Med 2005 (132)<br>n=48<br>Retrospective	*Impact on Diagnosis*<br>SPECT/CT: diagnosis of additional 2% PTA<br>SPECT/CT localization: additional 8% (including 2 ectopic)
Krausz et al, World J Surg 2006 (133)<br>n=36<br>Retrospective	*Preoperative localization*<br>SPECT/CT localized 14 PTAs (10 ectopic & 4 in distorted neck anatomy)<br>Role in planning surgery: 39% patients.
Lavely et al, J Nucl Med 2007 (134)<br>n=110<br>Prospective	*Comparison of various acquisition protocols*<br>Best diagnostic accuracy: early SPECT/CT + any delayed imaging
Neumann et al, J Nucl Med 2008 (135)<br>n=61<br>Prospective	*Preoperative localization*<br>SPECT/CT vs. SPECT: similar sensitivity (70% vs. 71%); higher specificity (96% vs. 48%)
Patel et al, Clin Radiol 2010 (136)<br>n=63<br>Retrospective	*Preoperative localization; compared to US*<br>Detectability rate: SPECT/CT 90% vs. US 64%.<br>Concordant findings on SPECT/CT & US: 59%.<br>US + SPECT/CT preoperative PTA localization: sensitivity 95%; accuracy 91%.
Pata et al, Thyroid 2010 (137)<br>n=33<br>Retrospective	*Diagnosis (specifically in multinodular goitre); compared to SPECT*<br>SPECT/CT localization (lateralization & neck quadrant): sensitivity 94 & 88%; specificity 93 & 96%; PPV 94 & 88%.<br>Mean time of surgery: SPECT/CT 38 min vs. SPECT 56 min.
Pata et al, Ann Surg Oncol 2011 (138)<br>n=55<br>Retrospective	*Cost-analysis for preoperative localization; compared to SPECT*<br>Mean time of surgery: SPECT/CT 36 min vs. SPECT 62 min.<br>SPECT/CT decrease in mean cost: 98.7 €.
Tokmak et al, Int J Clin Exp Med 2014 (139)<br>n=154<br>Retrospective	*Diagnosis & localization*<br>SPECT/CT detectability rate: 98%<br>Sensitivity increased mainly in small lesions.
Burall GG et al, Mol Imaging Radionucl Ther 2012 (140)<br>n=32	*Localization; compared to SPECT*<br>SPECT/CT 31/32 patients vs. SPECT 22/32<br>FN on SPECT: lesions <10mm.
Ciappuccini et al, Clin Nucl Med 2012 (141)<br>n=59	*Diagnosis & preoperative localization*<br>Diagnosis by 2-phase SPECT/CT in 66% patients<br>Correlation with serum Calcium and PTH values
Suh et al, Otolaryngol Head Neck Surg 2015 (142)<br>n=38<br>Retrospective	*Localization; compared with 4D-CT & US*<br>4D-CT outperformed US & SPECT/CT with unique anatomic data in 8% patients

Mandal et al, Laryngoscope 2015 (143)<br>n=75<br>Retrospective	*Diagnosis; dual-phase SPECT/CT*<br>Early-phase SPECT/CT 76% vs. late-phase 74%.<br>Early-phase localization: sensitivity 84%, specificity 89% (no improvement with dual-phase)
Koberstein et al, Can Assoc Radiol J 2016 (144)<br>n=88<br>Retrospective	*Preoperative localization (specifically for ectopic PTA) & correlation with serum PTH*<br>Localization: Similar accuracy & reliability for normal & ectopic PTAs (90 vs. 94%).<br>SPECT/CT accuracy correlates with serum PTH levels.
Barber et al, Head Neck 2016 (145)<br>n=259<br>Retrospective	*Cost-effectiveness of preoperative localization (combined with US)*<br>US + SPECT/CT lateralization: sensitivity 87%, PPV 99%.<br>Increased cost of US+SPECT/CT: 30% vs. SPECT/CT only
Keidar et al, Mol Imaging Biol 2017 (146)<br>n=88<br>Retrospective	*Preoperative localization (by Perrier criteria)*<br>SPECT/CT localization: accuracy 80%
Woods et al, Nucl Med Comm 2017 (147)<br>n=135<br>Retrospective	*Diagnosis & localization (combined with 123Iodine)*<br>DI-SPECT/CT detection & localization: sensitivity 95%, specificity 89%, PPV 97%, NPV 83%.<br>Accuracy: diagnosis 94%; localization 92%.
Sandquist et al, Clin Nucl Med 2017 (148)<br>n=249<br>Retrospective	*Preoperative localization; compared to SPECT*<br>SPECT/CT sensitivity 83%, specificity 96%,<br>SPECT/CT had fewer FPs (vs. SPECT)<br>Main advantage: PTAs < 210 mg.
Christakis et al, Eur J Radiol 2017 (149)<br>n=20	*Localization; compared to US & 4D-CT*<br>4D-CT + MIBI SPECT/CT: sensitivity 94% & accuracy 95%.<br>US + 4D-CT + MIBI SPECT/CT: sensitivity & accuracy 100%.
Cheng et al, Clin Nucl Med 2018 (150)<br>n=94	*Diagnosis & patient management*<br>Positive SPECT/CT predicted eligibility for surgery.

PTA – parathyroid adenoma
PTH – parathyroid hormone
PPV – positive predictive value; NPV – negative predictive value

Table 12: Literature evidence on the role of cardiac SPECT/CT

**Author/Source/Year**<br>**No. patients (n)**<br>**Clinical indications**<br>**Type of study**	**Aim & Results**
Schaap et al, J Cardiovasc Imaging, 2014, (151)<br>n=205<br>Diagnosis of CAD; CCTA<br>Prospective	*Incremental value of MP- SPECT/CCTA compared to SPECT and CCTA*<br>SPECT/CCTA had higher yield vs. stand-alone SPECT or CCTA in diagnosis of significant CAD
Schepis et al, Eur J Nucl Med Mol Imaging 2007 (152)<br>n=32<br>CT for AC & CCS	*Use of CT-AC & CCS measurements*<br>Attenuation maps derived from CT for CCS enable accurate AC
Rispler et al, Int J Cardiol 2013 (153)<br>n=53<br>123Iodine mIBG cardiac uptake quantitation<br>Prospective	*SPECT/CT quantitation of 123I-mIBG*<br>Total cardiac count measurements are feasible using the CT component for determining heart boundaries even in case of very low uptake
Rispler et al, JACC 2007 (154)<br>n=56<br>Physiologic significance of CAD; SPECT/CCTA<br>Prospective	*Physiologic significance of coronary lesions compared to CCTA*<br>SPECT/CCTA: improved specificity & PPV in patients with chest pain
Kennedy JA et al. J Nucl Cardiology 2017 (155)<br>n=312<br>CZT perfusion score data base; CZT-SPECT/CT for AC<br>Prospective	*Measurements of perfusion scores in CZT- and NaI MPI-SPECT/CT in AC & NAC studies*<br>Specific database for CZT cardiac SPECT is needed for accurate quantitative diagnostic perfusion scores
Schaap et al, Eur Heart J, Cardiovasc Imaging 2013 (156)<br>n=98<br>Performance in intermediate & high CAD likelihood<br>Prospective	*Performance of SPECT/CCTA compared to SPECT & CCTA*<br>SPECT/CCTA: superior for diagnosis of significant CAD
Pretorius et al, J Nucl Cardiol 2017 (157)<br>n= 1,103<br>Effect of respiratory motion & gender on MPI	Respiratory motion correction algorithms significantly reduce artefacts
Abdollahi et al, Eur J Radiol 2016 (158)<br>n=509<br>Radiation exposure estimates	CT dose parameters are very low and below the reference level.
Özdemir et al, Mol Imaging Radionucl Ther 2016. (159)<br>n=78<br>Prevalence of silent ischemia in (pre)diabetics<br>Prospective	SPECT/CT defined prevalence of silent ischemia & adverse events in asymptomatic (pre-)diabetics & predicted future CAD
Engbers et al, J Nucl Cardiol 2015; (160)<br>n=5,018<br>Algorithm for CAD diagnosis & dose reduction<br>Prospective	*Comparison of sequential algorithms for CAD diagnosis & dose reduction*<br>Stress first SPECT/CT: 50% of patients needed no additional testing.
Fiechter et al, Eur J Nucl Med Mol Imaging, 2011 (161)<br>n=66<br>Diagnostic accuracy of CZT-SPECT/CT<br>Prospective	*Compared to ICA*<br>CZT SPECT/CT: high accuracy for detection of angiographically identified lesions

Abadi et al, Eur J Radiol 2010 (162)<br>n=76<br>LV volumes & function measurements<br>Prospective	*Measurement of SPECT LV volumes & EF; compared to CCTA*<br>Caution when using SPECT and CT derived EF & volumes
Rispler et al, Eur J Nucl Med Mol Imaging 2011, (163)<br>n=90<br>Risk stratification in NSTE acute coronary syndrome<br>Retrospective	*SPECT/CCTA compared to TIMI risk score*<br>40% of high- & 16% of low TIMI-RS patients had hemodynamically significant lesions<br>Normal perfusion spared revascularization regardless of TIMI-RS.
Tamam et al, World J Nucl Med 2016, (164)<br>n=157<br>AC for MPI in (non)obese patients	*Diagnostic value of AC in inferior wall; compared to NAC; obese/non-obese patients*<br>Iterative reconstruction > FBP to correct diaphragm attenuation of inferior wall<br>AC with OSEM iterative reconstruction improves results of stress-only MPI, in particular in obese patients
Kennedy et al, J Nucl Med 2009, (165)<br>n=124<br>Mis-registration impact on AC-MPI quality<br>Retrospective	*Define SPECT/CT mis-registration with greatest impact on AC-MPI quality*<br>SPECT/CT: significant mis-registration in 23%, in direction of most severe artefacts in 16% studies (lateral & anterior walls; SPECT myocardium overlap on lungs on CT)
Koopman et al, Nucl Med Comm 2015, (166)<br>n=20<br>AC/NAC CZT-SPECT/CT processing protocols<br>Retrospective	*CZT-SPECT/CT processing protocols for detection of ischemia; AC compared to NAC*<br>Interoperator variations: 2.4±1.4% (NC) vs. 3.8±1.9% (AC)<br>CZT-SPECT/CT operator variations in MPI processing: significant & influence diagnosis, especially with AC
Matsuo et al, Annals Nucl Med 2015, (167)<br>n=40<br>AC/NAC acquisition protocols of 201Tl uptake<br>Retrospective	*Comparison of new & conventional acquisition protocols with/without AC*<br>Short IQ-SPECT acquisition: equivalent high image quality to conventional MPI

CAD – coronary artery disease
NSTE – non-ST-elevation
TIMI-RS – TIMI risk score
AC- attenuation correction; NAC – non-attenuated
CCS – coronary calcium score
CCTA – coronary CT angiography
MPI – myocardial perfusion imaging
CZT – Cadmium Zinc Tellurium
LV – left ventricle
FBP – filtered back projection

Table 13: Incidental findings on the CT component of the SPECT/CT

**MAJOR**	**MODERATE**	**MINOR**
**Head & neck**<br>Parietal meningioma<br>Orbital mass<br>Parotid mass<br>**Chest**<br>Pneumothorax[+]<br>Pulmonary embolism[+]<br>Solid pulmonary mass<br><br>**Abdomen**<br>Solid liver mass<br>Solid renal mass<br>Gall bladder mass<br>GIT mass<br>Pancreatic solid/cystic mass<br>Bilateral small kidneys<br>Adrenal mass<br>Retroperitoneal mass<br>**Pelvis**<br>Undescended testis<br>Ovarian cyst >5 cm<br><br>**Vascular**<br>Deep vein thrombosis[+]<br>Aortic aneurysm >5 cm[+]<br>Aortic dissection *<br><br>**Musculoskeletal**<br>Vertebral body destruction<br>Lytic bone lesions<br>Indeterminate sclerotic bone lesion<br><br>**Reticuloendothelial**<br>LN > 1.5 cm and/or multiple LNs	**Head & neck**<br>Thyroid incidentalomas<br><br>**Chest**<br>Pulmonary parenchymal opacity<br>Emphysema<br>Bronchiectasis<br>Pleural effusion<br>Cardiomegaly<br>Pericardial effusion<br>Breast nodule<br><br>**Abdomen**<br>Gallstone in common bile duct<br>Air in biliary tree<br>Absent kidney<br>Renal calculus<br>Hydronephrosis<br>Complex renal cyst<br>Splenomegaly<br>Bowel inflammation<br>Adrenal adenoma<br><br>**Pelvis**<br>Uterine mass<br>Uterine enlargement<br>Pelvic kidney<br>Ureteric calculus<br>Scrotal hydrocoelle<br>Prostate enlargement<br><br>**Vascular**<br>Aortic ectasia<br>Pulmonary artery dilatation<br>Signs of portal venous hypertension<br>Coronary artery calcification<br><br>**Reticuloendothelial**<br>LN > 1cm	**Head & neck**<br>Parathyroid adenoma<br><br>**Chest**<br>Calcified pulmonary nodule<br><br>**Abdomen**<br>Gallstones in gallbladder<br>Fatty liver<br>Hepatic cysts<br>Renal cysts<br>Renal atrophy<br>Appendicolith<br>Abdominal wall hernia<br>Umbilical hernia<br>Hiatus hernia<br><br>**Pelvis**<br>Lipoma<br>Bladder diverticulum<br>Bladder stone<br>Simple ovarian cyst<br>Uterine fibroids<br>Uterine calcifications<br>Bartholin's cysts<br><br>**Vascular**<br>Left-sided vena cava<br>Retroaortic left renal vein<br><br>**Musculoskeletal**<br>Muscle atrophy<br>Bone infarct<br>Degenerative spine changes

+: Notify referring physician

LN – lymph node

References

1. Giżewska A, Witkowska-Patena E, Osiecki S, Mazurek A, Stembrowicz-Nowakowska Z, Dziuk M. Utility of single-photon emission tomography/computed tomography for sentinel lymph node localization in breast cancer patients. Nucl Med Commun. 2017;38:493-9.
2. Borrelli P, Donswijk ML, Stokkel MP, Teixeira SC, van Tinteren H, Emiel JT, et al. Contribution of SPECT/CT for sentinel node localization in patients with ipsilateral breast cancer relapse. Eur J Nucl Med Mol Imaging. 2017;44:630-7.
3. Zetterlund L, Gabrielson S, Axelsson R, de Boniface J, Frisell J, Olsson A, et al. Impact of previous surgery on sentinel lymph node mapping: Hybrid SPECT/CT before and after a unilateral diagnostic breast excision. Breast. 2016;30:32-8.
4. Pouw B, Hellingman D, Kieft M, Vogel WV, van Os KJ, Rutgers E, et al. The hidden sentinel node in breast cancer: Reevaluating the role of SPECT/CT and tracer reinjection. Eur J Surg Oncol (EJSO). 2016;42:497-503.
5. Jimenez-Heffernan A, Ellmann A, Sado H, Huić D, Bal C, Parameswaran R, et al. Results of a prospective multicenter international atomic energy agency sentinel node trial on the value of SPECT/CT over planar imaging in various malignancies. J Nucl Med. 2015;56:1338-44.
6. Tomiguchi M, Yamamoto-Ibusuki M, Yamamoto Y, Fujisue M, Shiraishi S, Inao T, et al. Prediction of sentinel lymph node status using single-photon emission computed tomography (SPECT)/computed tomography (CT) imaging of breast cancer. Surgery Today. 2016;46:214-23.
7. Shima H, Kutomi G, Satomi F, Maeda H, Takamaru T, Kameshima H, et al. Risk of node metastasis of sentinel lymph nodes detected in level II/III of the axilla by single-photon emission computed tomography/computed tomography. Experim Therap Medicine. 2014;8:1447-52.
8. Kraft O, Havel M. Sentinel lymph nodes and planar scintigraphy and SPECT/CT in various types of tumours. Estimation of some factors influencing detection success. Nucl Med Review. 2013;16:17-25.
9. Yoneyama H, Tsushima H, Kobayashi M, Onoguchi M, Nakajima K, Kinuya S. Improved detection of sentinel lymph nodes in SPECT/CT images acquired using a low-to medium-energy general-purpose collimator. Clin Nucl Med. 2014;39:e1-e6.
10. Brouwer O, Vermeeren L, van der Ploeg I, Olmos RV, Loo C, Pereira-Bouda L, et al. Lymphoscintigraphy and SPECT/CT in multicentric and multifocal breast cancer: does each tumour have a separate drainage pattern? Eur J Nucl Med Mol Imaging. 2012;39:1137-43.
11. Uren R, Howman-Giles R, Chung D, Spillane A, Noushi F, Gillett D, et al. SPECT/CT scans allow precise anatomical location of sentinel lymph nodes in breast cancer and redefine lymphatic drainage from the breast to the axilla. The Breast. 2012;21:480-6.
12. Coffey J, Hill J. Breast sentinel node imaging with low-dose SPECT/CT. Nucl Med Commun. 2010;31:107-11.
13. Cheville AL, Das I, Srinivas S, Schuerman J, Velders L, Solin LJ, et al. A pilot study to assess the utility of SPECT/CT-based lymph node imaging to localize lymph nodes that drain the arm in patients undergoing treatment for breast cancer. Breast Cancer Res Treatm. 2009;116:531.
14. van der Ploeg IM, Olmos RAV, Kroon BB, Rutgers EJ, Nieweg OE. The hidden sentinel node and SPECT/CT in breast cancer patients. Eur J Nucl Med Mol Imaging. 2009;36:6-11.
15. Gallowitsch H, Kraschl P, Igerc I, Hussein T, Kresnik E, Mikosch P, et al. Sentinel node SPECT-CT in breast cancer. Can we expect any additional and clinically relevant information? Nuklearmedizin. 2007;46:252-6.
16. Lerman H, Lievshitz G, Zak O, Metser U, Schneebaum S, Even-Sapir E. Improved sentinel node identification by SPECT/CT in overweight patients with breast cancer. J Nucl Med. 2007;48:201-6.

17. Trinh BB, Chapman BC, Gleisner A, Kwak JJ, Morgan R, McCarter MD, et al. SPECT/CT adds distinct lymph node basins and influences radiologic findings and surgical approach for sentinel lymph node biopsy in head and neck melanoma. Ann Surg Oncol. 2018;25:1716-22.
18. Doepker MP, Yamamoto M, Applebaum MA, Patel NU, Montilla-Soler MJ, Sarnaik AA, et al. Comparison of Single-Photon Emission Computed Tomography–Computed Tomography (SPECT/CT) and conventional planar lymphoscintigraphy for sentinel node localization in patients with cutaneous malignancies. Ann Surg Oncol. 2017;24:355-61.
19. Stoffels I, Müller M, Geisel MH, Leyh J, Pöppel T, Schadendorf D, et al. Cost-effectiveness of preoperative SPECT/CT combined with lymphoscintigraphy vs. lymphoscintigraphy for sentinel lymph node excision in patients with cutaneous malignant melanoma. Eur J Nucl Med Mol Imaging. 2014;41:1723-31.
20. Zender C, Guo T, Weng C, Faulhaber P, Rezaee R. Utility of SPECT/CT for periparotid sentinel lymph node mapping in the surgical management of head and neck melanoma. Am J Otolaryngology. 2014;35:12-8.
21. Fairbairn N, Munson C, Khan ZA, Butterworth M. The role of hybrid SPECT/CT for lymphatic mapping in patients with melanoma. J Plast Reconstr Aesth Surgery. 2013;66:1248-55.
22. Vuthaluru S, Pushker N, Lokdarshi G, Kumar R, Bajaj MS, Kashyap S, et al. Sentinel lymph node biopsy in malignant eyelid tumor: hybrid single photon emission computed tomography/computed tomography and dual dye technique. Am J Ophthalmology. 2013;156:43-9. e2.
23. Stoffels I, Boy C, Pöppel T, Kuhn J, Klötgen K, Dissemond J, et al. Association between sentinel lymph node excision with or without preoperative SPECT/CT and metastatic node detection and disease-free survival in melanoma. JAMA. 2012;308:1007-14.
24. Kraft O, Havel M. Localisation of sentinel lymph nodes in patients with melanomas by planar lymphoscintigraphic and hybrid SPECT/CT imaging. Nucl Med Review. 2012;15:101-7.
25. Veenstra HJ, Klop WMC, Speijers MJ, Lohuis PJ, Nieweg OE, Hoekstra HJ, et al. Lymphatic drainage patterns from melanomas on the shoulder or upper trunk to cervical lymph nodes and implications for the extent of neck dissection. Ann Surg Oncology. 2012;19:3906-12.
26. Veenstra HJ, Vermeeren L, Olmos RAV, Nieweg OE. The additional value of lymphatic mapping with routine SPECT/CT in unselected patients with clinically localized melanoma. Ann Surgical Oncology. 2012;19:1018-23.
27. Nielsen KR, Chakera AH, Hesse B, Scolyer RA, Stretch JF, Thompson JF, et al. The diagnostic value of adding dynamic scintigraphy to standard delayed planar imaging for sentinel node identification in melanoma patients. Eur J Nucl Med Mol Imaging. 2011;38:1999.
28. Klode J, Poeppel T, Boy C, Mueller S, Schadendorf D, Körber A, et al. Advantages of preoperative hybrid SPECT/CT in detection of sentinel lymph nodes in cutaneous head and neck malignancies. J Eur Acad Derm Vener. 2011;25:1213-21.
29. Vermeeren L, Valdés Olmos RA, Klop WMC, van der Ploeg IM, Nieweg OE, Balm AJ, et al. SPECT/CT sentinel lymph node mapping in head and neck melanoma. Head & Neck. 2011;33:1-6.
30. van der Ploeg IM, Kroon BB, Olmos RAV, Nieweg OE. Evaluation of lymphatic drainage patterns to the groin and implications for the extent of groin dissection in melanoma patients. Ann Surg Oncol. 2009;16:2994.
31. van der Ploeg IM, Olmos RAV, Kroon BB, Wouters MW, van den Brekel MW, Vogel WV, et al. The yield of SPECT/CT for anatomical lymphatic mapping in patients with melanoma. Ann Surg Oncol. 2009;16:1537-42.
32. Ishihara T, Kaguchi A, Matsushita S, Shiraishi S, Tomiguchi S, Yamashita Y, et al. Management of sentinel lymph nodes in malignant skin tumors using dynamic

lymphoscintigraphy and the single-photon-emission computed tomography/computed tomography combined system. Internat J Clin Oncol. 2006;11:214-20.
33. Even-Sapir E, Lerman H, Lievshitz G, Khafif A, Fliss DM, Schwartz A, et al. Lymphoscintigraphy for sentinel node mapping using a hybrid SPECT/CT system. J Nucl Med. 2003;44:1413.
34. Avram AM, Esfandiari NH, Wong KK. Preablation ^{131}I SPECT/CT contributes to thyroid cancer risk stratification and 131I therapy planning. J Clin Endocr Metab 2015;100:1895-902.
35. Agrawal K, Bhattacharya A, Mittal BR. Role of single photon emission computed tomography/computed tomography in diagnostic iodine-131 scintigraphy before initial radioiodine ablation in differentiated thyroid cancer. Ind J Nucl Med. 2015;30:221.
36. Avram AM, Fig LM, Frey KA, Gross MD, Wong KK. Preablation 131-I scans with SPECT/CT in postoperative thyroid cancer patients: what is the impact on staging? J Clin Endocr Metab. 2013;98:1163-71.
37. Wong KK, Sisson JC, Koral KF, Frey KA, Avram AM. Staging of differentiated thyroid carcinoma using diagnostic ^{131}I SPECT/CT. AJR Am J Roentgenology. 2010;195:730-6.
38. Hassan FU, Mohan HK. Clinical utility of SPECT/CT imaging post-radioiodine therapy: does it enhance patient management in thyroid cancer? Eur Thyroid J. 2015;4:239-45.
39. Grewal RK, Michael Tuttle R, Fox J, Borkar S, Chou JF, Gonen M, et al. The effect of posttherapy ^{131}I SPECT/CT on risk classification and management of patients with differentiated thyroid cancer. J Nucl Med. 2010;51:1361.
40. Kohlfuerst S, Igerc I, Lobnig M, Gallowitsch H, Gomez-Segovia I, Matschnig S, et al. Posttherapeutic ^{131}I SPECT-CT offers high diagnostic accuracy when the findings on conventional planar imaging are inconclusive and allows a tailored patient treatment regimen. Eur J Nucl Med Mol Imaging. 2009;36:886.
41. Spanu A, Solinas ME, Chessa F, Sanna D, Nuvoli S, Madeddu G. ^{131}I SPECT/CT in the follow-up of differentiated thyroid carcinoma: incremental value versus planar imaging. J Nucl Med. 2009.50:184-90.
42. Wang H, Fu H-L, Li J-N, Zou R-J, Gu Z-H, Wu J-C. The role of single-photon emission computed tomography/computed tomography for precise localization of metastases in patients with differentiated thyroid cancer. Clin Imaging. 2009;33:49-54.
43. Wong KK, Zarzhevsky N, Cahill JM, Frey KA, Avram AM. Incremental value of diagnostic ^{131}I SPECT/CT fusion imaging in the evaluation of differentiated thyroid carcinoma. Am J Roentgenology. 2008;191:1785-94.
44. Tharp K, Israel O, Hausmann J, Bettman L, Martin W, Daitzchman M, et al. Impact of 131I-SPECT/CT images obtained with an integrated system in the follow-up of patients with thyroid carcinoma. Eur J Nucl Med Mol Imaging. 2004;31:1435-42.
45. Kunikowska J, Lewington V, Krolicki L. Optimizing somatostatin receptor imaging in patients with neuroendocrine tumors: the Impact of ^{99m}Tc-HYNICTOC SPECT/SPECT/CT versus ^{68}Ga-DOTATATE PET/CT upon clinical management. Clin Nucl Med. 2017;42:905-11.
46. Trogrlic M, Tezak S. Incremental value of ^{99m}Tc-HYNIC-TOC SPECT/CT over whole-body planar scintigraphy and SPECT in patients with neuroendocrine tumours. Nuklearmedizin. 2017;56:97-107.
47. de Camargo Etchebehere ECS, de Oliveira Santos A, Gumz B, Vicente A, Hoff PG, Corradi G, et al. ^{68}Ga-DOTATATE PET/CT, ^{99m}Tc-HYNIC-octreotide SPECT/CT, and whole-body MR imaging in detection of neuroendocrine tumors: a prospective trial. J Nucl Med. 2014;55:1598-604.
48. Spanu A, Schillaci O, Piras B, Calvisi DF, Falchi A, Danieli R, et al. Non-functioning gastroenteropancreatic (GEP) tumors: a ^{111}In-Pentetreotide SPECT/CT diagnostic study. Am J Nucl Med Mol Imaging. 2017;7:181-94.

49. Ait Boudaoud A, Verges B, Petit JM, Tatulashvili S, Cochet A, Humbert O. Uptake in the pancreatic uncinate process on the ^{111}In-octreotide scintigraphy: How to distinguish physiological from pathological uptake? Nucl Med Comm. 2017;38:737-43.
50. Ruf J, von Wedel F, Furth C, Denecke T, Stelter L, Steffen IG, et al. Significance of a single-time-point somatostatin receptor SPECT/multiphase CT protocol in the diagnostic work-up of gastroenteropancreatic neuroendocrine neoplasms. J Nucl Med. 2016;57:180-5.
51. Lee I, Paeng JC, Lee SJ, Shin CS, Jang JY, Cheon GJ, et al. Comparison of diagnostic sensitivity and quantitative indices between ^{68}Ga-DOTATOC PET/CT and ^{111}In-Pentetreotide SPECT/CT in neuroendocrine tumors: preliminary report. Nucl Med Mol Imaging. 2015;49:284-90.
52. Chiaravalloti A, Spanu A, Danieli R, Dore F, Piras B, Falchi A, et al. ^{111}In-Pentetreotide SPECT/CT in pulmonary carcinoid. Anticancer Research. 2015;35:4265-70.
53. Sainz-Esteban A, Olmos R, González-Sagrado M, González ML, Ruiz MÁ, García-Talavera P, et al. Contribution of ^{111}In-pentetreotide SPECT/CT imaging to conventional somatostatin receptor scintigraphy in the detection of neuroendocrine tumours. Nucl Med Comm. 2015;36:251-9.
54. Schreiter NF, Bartels A-M, Froeling V, Steffen I, Pape U-F, Beck A, et al. Searching for primaries in patients with neuroendocrine tumors (NET) of unknown primary and clinically suspected NET: evaluation of Ga-68 DOTATOC PET/CT and In-111 DTPA octreotide SPECT/CT. Radiol Oncol. 2014;48:339-47.
55. Apostolova I, Riethdorf S, Buchert R, Derlin T, Brenner W, Mester J, et al. SPECT/CT stabilizes the interpretation of somatostatin receptor scintigraphy findings: a retrospective analysis of inter-rater agreement. Ann Nucl Medicine. 2010;24:477-83.
56. Castaldi P, Rufini V, Treglia G, Bruno I, Perotti G, Stifano G, et al. Impact of ^{111}In-DTPA-octreotide SPECT/CT fusion images in the management of neuroendocrine tumours. La Radiologia Medica. 2008;113:1056-67.
57. Perri M, Erba P, Volterrani D, Lazzeri E, Boni G, Grosso M, et al. Octreo-SPECT/CT imaging for accurate detection and localization of suspected neuroendocrine tumors. Quart J Nucl Med Mol Imaging. 2008;52:323.
58. Hillel P, Van Beek E, Taylor C, Lorenz E, Bax N, Prakash V, et al. The clinical impact of a combined gamma camera/CT imaging system on somatostatin receptor imaging of neuroendocrine tumours. Clin Radiol. 2006;61:579-87.
59. Krausz Y, Keidar Z, Kogan I, Even-Sapir E, Bar-Shalom R, Engel A, et al. SPECT/CT hybrid imaging with ^{111}In-pentetreotide in assessment of neuroendocrine tumours. Clin Endocr. 2003;59:565-73.
60. Chang CA, Pattison DA, Tothill RW, Kong G, Akhurst TJ, Hicks RJ, et al. ^{68}Ga-DOTATATE and ^{18}F-FDG PET/CT in paraganglioma and pheochromocytoma: utility, patterns and heterogeneity. Cancer Imaging. 2016;16:22.
61. Kroiss AS, Uprimny C, Shulkin BL, Frech A, Tilg H, Gasser RW, et al. Compared to ^{123}I-MIBG SPECT/CT, ^{18}F-DOPA PET/CT provides accurate tumor extent in patients with extra-adrenal paraganglioma. Ann Nucl Med. 2017;31:357-65.
62. Nakamoto R, Nakamoto Y, Ishimori T, Togashi K. Clinical significance of quantitative ^{123}I-MIBG SPECT/CT analysis of pheochromocytoma and paraganglioma. Clin Nucl Med. 2016;41:e465-e72.
63. Kroiss A, Shulkin BL, Uprimny C, Frech A, Gasser RW, Url C, et al. ^{68}Ga-DOTATOC PET/CT provides accurate tumour extent in patients with extraadrenal paraganglioma compared to ^{123}I-MIBG SPECT/CT. Eur J Nucl Med Mol Imaging. 2015;42:33-41.
64. Derlin T, Busch JD, Wisotzki C, Schoennagel BP, Bannas P, Papp L, et al. Intraindividual comparison of ^{123}I-mIBG SPECT/MRI, ^{123}I-mIBG SPECT/CT, and MRI for the detection of adrenal

pheochromocytoma in patients with elevated urine or plasma catecholamines. Clin Nucl Med. 2013;38:e1-e6.
65. Fukuoka M, Taki J, Mochizuki T, Kinuya S. Comparison of diagnostic value of I-123 MIBG and high-dose I-131 MIBG scintigraphy including incremental value of SPECT/CT over planar image in patients with malignant pheochromocytoma/paraganglioma and neuroblastoma. Clin Nucl Med. 2011;36:1-7.
66. Meyer-Rochow GY, Schembri GP, Benn DE, Sywak MS, Delbridge LW, Robinson BG, et al. The utility of metaiodobenzylguanidine single photon emission computed tomography/computed tomography (MIBG SPECT/CT) for the diagnosis of pheochromocytoma. Ann Surg Oncol. 2010;17:392-400.
67. Zhao Z, Li L, Li F, Zhao L. Single photon emission computed tomography/spiral computed tomography fusion imaging for the diagnosis of bone metastasis in patients with known cancer. Skel Radiol. 2010;39:147.
68. Palmedo H, Marx C, Ebert A, Kreft B, Ko Y, Türler A, et al. Whole-body SPECT/CT for bone scintigraphy: diagnostic value and effect on patient management in oncological patients. Eur J Nucl Med Mol Imaging. 2014;41:59-67.
69. Zhang Y, Shi H, Li B, Cai L, Gu Y, Xiu Y. The added value of SPECT/spiral CT in patients with equivocal bony metastasis from hepatocellular carcinoma. Nuklearmedizin. 2015;54:255-61.
70. Haraldsen A, Bluhme H, Røhl L, Pedersen EM, Jensen AB, Hansen EB, et al. Single photon emission computed tomography (SPECT) and SPECT/low-dose computerized tomography did not increase sensitivity or specificity compared to planar bone scintigraphy for detection of bone metastases in advanced breast cancer. Clin Physiol Funct Imaging. 2016;36:40-6.
71. Jambor I, Kuisma A, Ramadan S, Huovinen R, Sandell M, Kajander S, et al. Prospective evaluation of planar bone scintigraphy, SPECT, SPECT/CT, ^{18}F-NaF PET/CT and whole body 1.5T MRI, including DWI, for the detection of bone metastases in high risk breast and prostate cancer patients: SKELETA clinical trial. Acta Oncol. 2016;55:59-67.
72. Fonager RF, Zacho HD, Langkilde NC, Fledelius J, Ejlersen JA, Haarmark C, et al. Diagnostic test accuracy study of ^{18}F-sodium fluoride PET/CT, ^{99m}Tc-labelled diphosphonate SPECT/CT, and planar bone scintigraphy for diagnosis of bone metastases in newly diagnosed, high-risk prostate cancer. Am J Nucl Med Mol Imaging. 2017;7:218-27.
73. Mahaletchumy T, AbAziz A. Incremental value of single-photon emission computed tomography-computed tomography for characterization of skeletal lesions in breast cancer patients. World J Nucl Med. 2017;16:303.
74. Dittmann H, Kopp D, Kupferschlaeger J, Feil D, Groezinger G, Syha R, et al. A prospective study of quantitative SPECT/CT for evaluation of lung shunt fraction before SIRT of liver tumors. J Nucl Med. 2018;59:1366-72.
75. Yue J, Mauxion T, Reyes DK, Lodge MA, Hobbs RF, Rong X, et al. Comparison of quantitative Y-90 SPECT and non-time-of-flight PET imaging in post-therapy radioembolization of liver cancer. Med Physics. 2016;43:5779-90.
76. Erxleben C, Scheurig-Münkler C, Geisel D, Hamm B, Gebauer B, Powerski MJ. Hepatopulmonary shunting after surgical, interventional and systemic therapy in patients with liver malignancies scheduled for radioembolization. Acta Radiol. 2016;57:908-13.
77. Theysohn JM, Ruhlmann M, Müller S, Dechene A, Best J, Haubold J, et al. Radioembolization with Y-90 glass microspheres: do we really need SPECT-CT to identify extrahepatic shunts? PLoS One. 2015;10:e0137587.
78. Gates VL, Singh N, Lewandowski RJ, Spies S, Salem R. Intraarterial hepatic SPECT/CT imaging using ^{99m}Tc-macroaggregated albumin in preparation for radioembolization. J Nucl Med. 2015;56:1157-62.

79. Ilhan H, Goritschan A, Paprottka P, Jakobs TF, Fendler WP, Bartenstein P, et al. Systematic evaluation of tumoral ^{99m}Tc-MAA uptake using SPECT and SPECT/CT in 502 patients before ^{90}Y radioembolization. J Nucl Med. 2015;56:333-8.
80. Spreafico C, Morosi C, Maccauro M, Romito R, Lanocita R, Civelli EM, et al. Intrahepatic flow redistribution in patients treated with radioembolization. Cardiovasc Intervent Radiol. 2015;38:322-8.
81. van den Hoven AF, Smits ML, de Keizer B, van Leeuwen MS, van den Bosch MA, Lam MG. Identifying aberrant hepatic arteries prior to intra-arterial radioembolization. Cardiovasc Intervent Radiol. 2014;37:1482-93.
82. Zade AA, Rangarajan V, Purandare NC, Shah SA, Agrawal AR, Kulkarni SS, et al. ^{90}Y microsphere therapy: does ^{90}Y PET/CT imaging obviate the need for ^{90}Y Bremsstrahlung SPECT/CT imaging? Nucl Med Comm. 2013;34:1090-6.
83. Padia SA, Alessio A, Kwan SW, Lewis DH, Vaidya S, Minoshima S. Comparison of positron emission tomography and bremsstrahlung imaging to detect particle distribution in patients undergoing yttrium-90 radioembolization for large hepatocellular carcinomas or associated portal vein thrombosis. J Vasc Interv Radiol. 2013;24:1147-53.
84. Burgmans M, Too C, Kao Y, Goh A, Chow P, Tan B, et al. Computed tomography hepatic arteriography has a hepatic falciform artery detection rate that is much higher than that of digital subtraction angiography and 99mTc-MAA SPECT/CT: Implications for planning ^{90}Y radioembolization? Eur J Radiol. 2012;81:3979-84.
85. Ahmadzadehfar H, Muckle M, Sabet A, Wilhelm K, Kuhl C, Biermann K, et al. The significance of bremsstrahlung SPECT/CT after yttrium-90 radioembolization treatment in the prediction of extrahepatic side effects. Eur J Nucl Med Mol Imaging. 2012;39:309-15.
86. Lauenstein T, Heusner T, Hamami M, Ertle J, Schlaak J, Gerken G, et al. Radioembolization of hepatic tumors: flow redistribution after the occlusion of intrahepatic arteries. RöFo. 2011;183:1058-64.
87. Hamami ME, Poeppel TD, Muller S, Heusner T, Bockisch A, Hilgard P, et al. SPECT/CT with ^{99m}Tc-MAA in radioembolization with ^{90}Y microspheres in patients with hepatocellular cancer. J Nucl Med. 2009;50:688-92.
88. Denecke T, Rühl R, Hildebrandt B, Stelter L, Grieser C, Stiepani H, et al. Planning transarterial radioembolization of colorectal liver metastases with Yttrium 90 microspheres: evaluation of a sequential diagnostic approach using radiologic and nuclear medicine imaging techniques. Eur Radiology. 2008;18:892-902.
89. Russo VM, Dhawan RT, Baudracco I, Dharmarajah N, Lazzarino AI, Casey AT. Hybrid bone SPECT/CT imaging in evaluation of chronic low back pain: correlation with facet joint arthropathy. World Neurosurg. 2017;107:732-8.
90. Hudyana H, Maes A, Vandenberghe T, Fidlers L, Sathekge M, Nicolai D, et al. Accuracy of bone SPECT/CT for identifying hardware loosening in patients who underwent lumbar fusion with pedicle screws. Eur J Nucl Med Mol Imaging. 2016;43:349-54.
91. Sumer J, Schmidt D, Ritt P, Lell M, Forst R, Kuwert T, et al. SPECT/CT in patients with lower back pain after lumbar fusion surgery. Nucl Med Comm. 2013;34:964-70.
92. Ha S, Hong SH, Paeng JC, Lee DY, Cheon GJ, Arya A, et al. Comparison of SPECT/CT and MRI in diagnosing symptomatic lesions in ankle and foot pain patients: diagnostic performance and relation to lesion type. PLoS One. 2015;10:e0117583.
93. Chicklore S, Gnanasegaran G, Vijayanathan S, Fogelman I. Potential role of multislice SPECT/CT in impingement syndrome and soft-tissue pathology of the ankle and foot. Nucl Med Comm. 2013;34:130-9.

94. Huellner MW, Bürkert A, Strobel K, Lago MdSP, Werner L, Hug U, et al. Imaging non-specific wrist pain: interobserver agreement and diagnostic accuracy of SPECT/CT, MRI, CT, bone scan and plain radiographs. PloS One. 2013;8:e85359.
95. Schleich FS, Schürch M, Huellner MW, Hug U, von Wartburg U, Strobel K, et al. Diagnostic and therapeutic impact of SPECT/CT in patients with unspecific pain of the hand and wrist. EJNMMI research. 2012;2:53.
96. Dobrindt O, Amthauer H, Krueger A, Ruf J, Wissel H, Grosser OS, et al. Hybrid SPECT/CT for the assessment of a painful hip after uncemented total hip arthroplasty. BMC Med Imag. 2015;15:18.
97. Chew CG, Lewis P, Middleton F, van den Wijngaard R, Deshaies A. Radionuclide arthrogram with SPECT/CT for the evaluation of mechanical loosening of hip and knee prostheses. Ann Nucl Med. 2010;24:735-43.
98. Slevin O, Schmid FA, Schiapparelli F, Rasch H, Hirschmann MT. Increased in vivo patellofemoral loading after total knee arthroplasty in resurfaced patellae. Knee Surg Sports Traumatol Arthrosc. 2018;26:1805-10.
99. Horger M, Eschmann SM, Pfannenberg C, Storek D, Dammann F, Vonthein R, et al. The value of SPET/CT in chronic osteomyelitis. Eur J Nucl Med Mol Imaging. 2003;30:1665-73.
100. Filippi L, Schillaci O, Santoni R, Manni C, Danieli R, Simonetti G. Usefulness of SPECT/CT with a hybrid camera for the functional anatomical mapping of primary brain tumors by [Tc99m] tetrofosmin. Cancer Biother Radiophar. 2006;21:41-8.
101. Bar-Shalom R, Yefremov N, Guralnik L, Keidar Z, Engel A, Nitecki S, et al. SPECT/CT using ^{67}Ga and ^{111}In-labeled leukocyte scintigraphy for diagnosis infection. J Nuc Med. 2006;47:587-94.
102. Horger M, Eschmann SM, Pfannenberg C, Storek D, Vonthein R, Claussen CD, et al. Added value of SPECT/CT in patients suspected of having bone infection: preliminary results. Arch Orthop Traum Surg. 2007;127:211-21.
103. Sathekge M, Garcia-Perez O, Paez D, El-Haj N, Kain-Godoy T, Lawal I, et al. Molecular imaging in musculoskeletal infections with ^{99m}Tc-UBI 29-41 SPECT/CT. Ann Nucl Med. 2018;32:54-9.
104. Djekidel M, Brown RK, Piert M. Benefits of hybrid SPECT/CT for ^{111}In-Oxine-and ^{99m}Tc-Hexamethylpropylene Amine Oxime-labeled leukocyte imaging. Clin Nucl Med. 2011;36:e50-e6.
105. Filippi L, Uccioli L, Giurato L, Schillaci O. Diabetic foot infection: usefulness of SPECT/CT for ^{99m}Tc-HMPAO-labeled leukocyte imaging. J Nucl Med. 2009;50:1042-6.
106. Heiba SI, Kolker D, Mocherla B, Kapoor K, Jiang M, Son H, et al. The optimized evaluation of diabetic foot infection by dual isotope SPECT/CT imaging protocol. J Foot Ankle Surg. 2010;49:529-36.
107. Erdman WA, Buethe J, Bhore R, Ghayee HK, Thompson C, Maewal P, et al. Indexing severity of diabetic foot infection with ^{99m}Tc-WBC SPECT/CT hybrid imaging. Diabetes Care. 2012;35:1826-31.
108. Aslangul E, M'bemba J, Caillat-Vigneron N, Coignard S, Larger E, Boitard C, et al. Diagnosing diabetic foot osteomyelitis in patients without signs of soft tissue infection by coupling hybrid ^{67}Ga SPECT/CT with bedside percutaneous bone puncture. Diabetes Care. 2013; 36:2203-10.
109. Heiba S, Kolker D, Ong L, Sharma S, Travis A, Teodorescu V, et al. Dual-isotope SPECT/CT impact on hospitalized patients with suspected diabetic foot infection: saving limbs, lives, and resources. Nucl Med Com. 2013;34:877-84.
110. Vouillarmet J, Morelec I, Thivolet C. Assessing diabetic foot osteomyelitis remission with white blood cell SPECT/CT imaging. Diab Med. 2014;31:1093-9.

111. La JF, Bhavan K, Lam K, Van SA, Erdman W, Lavery LA, et al. Comparison between Tc-99m WBC SPECT/CT and MRI for the diagnosis of biopsy-proven diabetic foot osteomyelitis. Wounds: a compendium of clinical research and practice. 2016;28:271-8.
112. Lazaga F, Van Asten SA, Nichols A, Bhavan K, La Fontaine J, Oz OK, et al. Hybrid imaging with ^{99m}Tc-WBC SPECT/CT to monitor the effect of therapy in diabetic foot osteomyelitis. Int Wound J. 2016;13:1158-60.
113. Vouillarmet J, Moret M, Morelec I, Michon P, Dubreuil J. Application of white blood cell SPECT/CT to predict remission after a 6 or 12 week course of antibiotic treatment for diabetic foot osteomyelitis. Diabetologia. 2017;60:2486-94.
114. Fuster D, Solà O, Soriano A, Monegal A, Setoain X, Tomás X, et al. A prospective study comparing whole-body FDG PET/CT to combined planar bone scan with ^{67}Ga SPECT/CT in the diagnosis of spondylodiskitis. Clin Nucl Med. 2012;37:827-32.
115. Tamm AS, Abele JT. Bone and gallium single-photon emission computed tomography-computed tomography is equivalent to magnetic resonance imaging in the diagnosis of infectious spondylodiscitis: A retrospective study. Can Assoc Radiol J. 2017;68:41-6.
116. Lazzeri E, Erba P, Perri M, Doria R, Tascini C, Mariani G. Clinical impact of SPECT/CT with In-111 biotin on the management of patients with suspected spine infection. Clin Nucl Med. 2010;35:12-7.
117. Chakraborty D, Bhattacharya A, Gupta AK, Panda NK, Das A, Mittal BR. Skull base osteomyelitis in otitis externa: The utility of triphasic and single photon emission computed tomography/computed tomography bone scintigraphy. Indian journal of nuclear medicine. Ind J Nucl Med. 2013;28:65-9.
118. Sharma P, Agarwal KK, Kumar S, Singh H, Bal C, Malhotra A, et al. Utility of ^{99m}Tc-MDP hybrid SPECT-CT for diagnosis of skull base osteomyelitis: comparison with planar bone scintigraphy, SPECT, and CT. Jap J Radiol. 2013;31:81-8.
119. Bolouri C, Merwald M, Huellner MW, Veit-Haibach P, Kuttenberger J, Pérez-Lago M, et al. Performance of orthopantomography, planar scintigraphy, CT alone and SPECT/CT in patients with suspected osteomyelitis of the jaw. Eur J Nucl Med Mol Imaging. 2013;40:411-7.
120. Graute V, Feist M, Lehner S, Haug A, Müller PE, Bartenstein P, et al. Detection of low-grade prosthetic joint infections using ^{99m}Tc-antigranulocyte SPECT/CT: initial clinical results. Eur J Nucl Med Mol Imaging. 2010;37:1751-9.
121. Kim HO, Na SJ, Oh SJ, Jung BS, Lee S-H, Chang JS, et al. Usefulness of adding SPECT/CT to ^{99m}Tc-hexamethylpropylene amine oxime (HMPAO)-labeled leukocyte imaging for diagnosing prosthetic joint infections. J Comp Assist Tomog. 2014;38:313-9.
122. Lou L, Alibhai KN, Winkelaar GB, Turnbull RG, Hoskinson ME, Warshawski R, et al. ^{99m}Tc-WBC scintigraphy with SPECT/CT in the evaluation of arterial graft infection. Nucl Med Comm. 2010;31:411-6.
123. Khaja MS, Sildiroglu O, Hagspiel K, Rehm PK, Cherry KJ, Turba UC. Prosthetic vascular graft infection imaging. Clin Imaging. 2013;37:239-44.
124. Erba P, Leo G, Sollini M, Tascini C, Boni R, Berchiolli R, et al. Radiolabelled leucocyte scintigraphy versus conventional radiological imaging for the management of late, low-grade vascular prosthesis infections. Eur J Nucl Med Mol Imaging. 2014;41:357-68.
125. Erba PA, Conti U, Lazzeri E, Sollini M, Doria R, De Tommasi SM, et al. Added value of ^{99m}Tc-HMPAO-labeled leukocyte SPECT/CT in the characterization and management of patients with infectious endocarditis. J Nucl Med. 2012;53:1235-43.
126. Lauridsen TK, Iversen KK, Ihlemann N, Hasbak P, Loft A, Berthelsen AK, et al. Clinical utility of ^{18}F-FDG positron emission tomography/computed tomography scan vs. ^{99m}Tc-HMPAO white

blood cell single-photon emission computed tomography in extra-cardiac work-up of infective endocarditis. Int J Cardiovasc Imaging. 2017;33:751-60.
127. Litzler P-Y, Manrique A, Etienne M, Salles A, Edet-Sanson A, Vera P, et al. Leukocyte SPECT/CT for detecting infection of left-ventricular-assist devices: preliminary results. J Nucl Med. 2010;51:1044-8.
128. Erba PA, Sollini M, Conti U, Bandera F, Tascini C, De Tommasi SM, et al. Radiolabeled WBC scintigraphy in the diagnostic workup of patients with suspected device-related infections. JACC: Cardiovasc Imaging. 2013;6:1075-86.
129. Heiba SI, Stempler L, Sullivan T, Kolker D, Kostakoglu L. The ideal dual-isotope imaging combination in evaluating patients with suspected infection of pelvic pressure ulcers. Nucl Med Comm. 2017;38:129-34.
130. Hung B-T, Wang P-W, Su Y-J, Huang W-C, Chang Y-H, Huang S-H, et al. The efficacy of ^{18}F-FDG PET/CT and ^{67}Ga SPECT/CT in diagnosing fever of unknown origin. Int J Inf Dis. 2017;62:10-7.
131. Nowosinska E, Navalkissoor S, Quigley AM, Buscombe JR. Is there a role for Gallium-67 citrate SPECT/CT, in patients with renal impairment or who are renal transplant recipients, in identifying and localizing suspected infection? World J Nucl Med. 2015;14:184-8.
132. Gayed IW, Kim EE, Broussard WF, Evans D, Lee J, Broemeling LD, et al. The value of ^{99m}Tc-sestamibi SPECT/CT over conventional SPECT in the evaluation of parathyroid adenomas or hyperplasia. J Nucl Med. 2005;46:248-52.
133. Krausz Y, Bettman L, Guralnik L, Yosilevsky G, Keidar Z, Bar-Shalom R, et al. Technetium-99m-MIBI SPECT/CT in primary hyperparathyroidism. World J Surg. 2006;30:76-83.
134. Lavely WC, Goetze S, Friedman KP, Leal JP, Zhang Z, Garret-Mayer E, et al. Comparison of SPECT/CT, SPECT, and planar imaging with single- and dual-phase ^{99m}Tc-sestamibi parathyroid scintigraphy. J Nucl Med. 2007;48:1084-9.
135. Neumann D, Obuchowski N, Difilippo F. Preoperative ^{123}I/^{99m}Tc-sestamibi subtraction SPECT and SPECT/CT in primary hyperparathyroidism. J Nucl Med. 2008;49:2012-7.
136. Patel C, Salahudeen H, Lansdown M, Scarsbrook A. Clinical utility of ultrasound and ^{99m}Tc-sestamibi SPECT/CT for preoperative localization of parathyroid adenoma in patients with primary hyperparathyroidism. Clin Radiol. 2010;65:278-87.
137. Pata G, Casella C, Besuzio S, Mittempergher F, Salerni B. Clinical appraisal of 99m technetium-sestamibi SPECT/CT compared to conventional SPECT in patients with primary hyperparathyroidism and concomitant nodular goiter. Thyroid. 2010;20:1121-7.
138. Pata G, Casella C, Magri GC, Lucchini S, Panarotto MB, Crea N, et al. Financial and clinical implications of low-energy CT combined with 99m Technetium-sestamibi SPECT for primary hyperparathyroidism. Ann Surg Oncology. 2011;18:2555-63.
139. Tokmak H, Demirkol MO, Alagöl F, Tezelman S, Terzioglu T. Clinical impact of SPECT-CT in the diagnosis and surgical management of hyper-parathyroidism. International journal of clinical and experimental medicine. 2014;7:1028-34.
140. Bural GG, Muthukrishnan A, Oborski MJ, Mountz JM. Improved benefit of SPECT/CT compared to SPECT alone for the accurate localization of endocrine and neuroendocrine tumors. Mol Imag Radionucl Ther. 2012;21:91-6.
141. Ciappuccini R, Morera J, Pascal P, Rame J-P, Heutte N, Aide N, et al. Dual-phase 99mTc sestamibi scintigraphy with neck and thorax SPECT/CT in primary hyperparathyroidism: a single-institution experience. Clin Nucl Med. 2012;37:223-8.
142. Suh YJ, Choi JY, Kim S-j, Chun IK, Yun TJ, Lee KE, et al. Comparison of 4D CT, ultrasonography, and ^{99m}Tc-sestamibi SPECT/CT in localizing single-gland primary hyperparathyroidism. Otolaryng – Head Neck Surg. 2015;152:438-43.

143. Mandal R, Muthukrishnan A, Ferris RL, de Almeida JR, Duvvuri U. Accuracy of early-phase versus dual-phase single-photon emission computed tomography/computed tomography in the localization of parathyroid disease. Laryngoscope. 2015;125:1496-501.
144. Koberstein W, Fung C, Romaniuk K, Abele JT. Accuracy of dual phase single-photon emission computed tomography/computed tomography in primary hyperparathyroidism: correlation with serum parathyroid hormone levels. Can Assoc Radiol J. 2016;67:115-21.
145. Barber B, Moher C, Côté D, Fung E, O'connell D, Dziegielewski P, et al. Comparison of single photon emission CT (SPECT) with SPECT/CT imaging in preoperative localization of parathyroid adenomas: A cost-effectiveness analysis. Head & Neck. 2016;38(S1):E2062-E5.
146. Keidar Z, Solomonov E, Karry R, Frenkel A, Israel O, Mekel M. Preoperative [^{99m}Tc]MIBI SPECT/CT interpretation criteria for localization of parathyroid adenomas - Correlation with surgical findings. Mol Imag Biol. 2017;19:265-70.
147. Woods A-M, Bolster AA, Han S, Poon F-W, Colville D, Shand J, et al. Dual-isotope subtraction SPECT-CT in parathyroid localization. Nucl Med Comm. 2017;38:1047-54.
148. Sandqvist P, Nilsson I-L, Grybäck P, Sanchez-Crespo A, Sundin A. SPECT/CT's advantage for preoperative localization of small parathyroid adenomas in primary hyperparathyroidism. Clin Nucl Med. 2017;42:e109-e14.
149. Christakis I, Vu T, Chuang HH, Fellman B, Figueroa AMS, Williams MD, et al. The diagnostic accuracy of neck ultrasound, 4D-Computed tomographyand sestamibi imaging in parathyroid carcinoma. Eur J Radiol. 2017;95:82-8.
150. Cheng Z, Zou S, Peng D, Zhang G, Zhu X. Prognostic value of ^{99m}Tc-Sestamibi parathyroid scintigraphy in predicting future surgical eligibility in patients with asymptomatic primary hyperparathyroidism. Clin Nucl Med. 2018;43:151-4.
151. Schaap J, de Groot JA, Nieman K, Meijboom WB, Boekholdt SM, Kauling RM, et al. Added value of hybrid myocardial perfusion SPECT and CT coronary angiography in the diagnosis of coronary artery disease. Eur Heart J - Cardiovasc Imaging. 2014;15:1281-8.
152. Schepis T, Gaemperli O, Koepfli P, Rüegg C, Burger C, Leschka S, et al. Use of coronary calcium score scans from stand-alone multislice computed tomography for attenuation correction of myocardial perfusion SPECT. Eur J Nucl Med Mol Imaging. 2007;34:11-9.
153. Rispler S, Frenkel A, Kuptzov E, Brodov Y, Israel O, Keidar Z. Quantitative ^{123}I-MIBG SPECT/CT assessment of cardiac sympathetic innervation - A new diagnostic tool for heart failure. Int J Cardiol. 2013;168:1556-8.
154. Rispler S, Keidar Z, Ghersin E, Roguin A, Soil A, Dragu R, et al. Integrated single-photon emission computed tomography and computed tomography coronary angiography for the assessment of hemodynamically significant coronary artery lesions. JACC. 2007;49:1059-67.
155. Kennedy JA, Brodov Y, Weinstein AL, Israel O, Frenkel A. The effect of CT-based attenuation correction on the automatic perfusion score of myocardial perfusion imaging using a dedicated cardiac solid-state CZT SPECT/CT. J Nucl Cardiol. 2017:1-10.
156. Schaap J, Kauling RM, Boekholdt SM, Nieman K, Meijboom WB, Post MC, et al. Incremental diagnostic accuracy of hybrid SPECT/CT coronary angiography in a population with an intermediate to high pre-test likelihood of coronary artery disease. Eur Heart J – Cardiovasc Imaging. 2013;14:642-9.
157. Pretorius PH, Johnson KL, Dahlberg ST, King MA. Investigation of the physical effects of respiratory motion compensation in a large population of patients undergoing Tc-99m cardiac perfusion SPECT/CT stress imaging. J Nucl Cardiol. 2017.2350-017-0890-3.
158. Abdollahi H, Shiri I, Salimi Y, Sarebani M, Mehdinia R, Deevband MR, et al. Radiation dose in cardiac SPECT/CT: An estimation of SSDE and effective dose. Eur J Radiol. 2016;85:2257-61.

159. Özdemir E, Polat ŞB, Yıldırım N, Türkölmez Ş, Ersoy R, Durmaz T, et al. Evaluation of silent myocardial ischemia with Single-Photon Emission Computed Tomography/Computed Tomography in asymptomatic subjects with diabetes and pre-diabetes. Mol Imag Radionucl Ther. 2016;25:70.
160. Engbers EM, Timmer JR, Ottervanger JP, Mouden M, Oostdijk AH, Knollema S, et al. Sequential SPECT/CT imaging for detection of coronary artery disease in a large cohort: evaluation of the need for additional imaging and radiation exposure. J Nucl Cardiol. 2017;24:212-23.
161. Fiechter M, Ghadri JR, Kuest SM, Pazhenkottil AP, Wolfrum M, Nkoulou RN, et al. Nuclear myocardial perfusion imaging with a novel cadmium-zinc-telluride detector SPECT/CT device: first validation versus invasive coronary angiography. Eur J Nucl Med Mol Imaging. 2011;38:2025-36.
162. Abadi S, Brook OR, Rispler S, Frenkel A, Engel A, Keidar Z. Hybrid cardiac SPECT/64-slice CTA-derived LV function parameters: correlation and reproducibility assessment. Eur J Radiol. 2010;75:154-8.
163. Rispler S, Aronson D, Abadi S, Roguin A, Engel A, Beyar R, et al. Integrated SPECT/CT for assessment of haemodynamically significant coronary artery lesions in patients with acute coronary syndrome. Eur J Nucl Med Mol Imaging. 2011;38:1917-25.
164. Tamam M, Mulazimoglu M, Edis N, Ozpacaci T. The value of attenuation correction in hybrid cardiac SPECT/CT on inferior wall according to body mass index. World J Nucl Med. 2016;15:18-23.
165. Kennedy JA, Israel O, Frenkel A. Directions and magnitudes of misregistration of CT attenuation-corrected myocardial perfusion studies: incidence, impact on image quality, and guidance for reregistration. J Nucl Med. 2009;50:1471-8.
166. Koopman D, van Dalen JA, Slump CH, Lots D, Timmer JR, Jager PL. Impact of image processing in the detection of ischaemia using CZT-SPECT/CT. Nucl Med Comm. 2015;36:60-8.
167. Matsuo S, Nakajima K, Onoguchi M, Wakabayash H, Okuda K, Kinuya S. Nuclear myocardial perfusion imaging using thallium-201 with a novel multifocal collimator SPECT/CT: IQ-SPECT versus conventional protocols in normal subjects. Ann Nucl Med. 2015;29:452-9.
